# A Novel Analog Reasoning Paradigm: New Insights in Intellectually Disabled Patients

**DOI:** 10.1371/journal.pone.0149717

**Published:** 2016-02-26

**Authors:** Aurore Curie, Amandine Brun, Anne Cheylus, Anne Reboul, Tatjana Nazir, Gérald Bussy, Karine Delange, Yves Paulignan, Sandra Mercier, Albert David, Stéphanie Marignier, Lydie Merle, Bénédicte de Fréminville, Fabienne Prieur, Michel Till, Isabelle Mortemousque, Annick Toutain, Eric Bieth, Renaud Touraine, Damien Sanlaville, Jamel Chelly, Jian Kong, Daniel Ott, Behrouz Kassai, Nouchine Hadjikhani, Randy L. Gollub, Vincent des Portes

**Affiliations:** 1 Centre de Référence, Déficiences Intellectuelles de Causes Rares, Hôpital Femme Mère Enfant, Hospices Civils de Lyon, Bron, France; 2 CNRS UMR 5304, L2C2, Institut des Sciences Cognitives, Bron, France; 3 Université de Lyon, Faculté de médecine Lyon Sud—Charles Mérieux, Lyon, France; 4 Athinoula A. Martinos Center for Biomedical Imaging, Massachusetts General Hospital, Charlestown, MA, United States of America; 5 EPICIME-CIC 1407/Inserm, UMR5558, LBBE, Université de Lyon, France; 6 Génétique, Centre Hospitalier Universitaire, Nantes, France; 7 Centre de Recherche en Neurosciences de Lyon, INSERM U1028, CNRS UMR5292, CH le Vinatier, France; 8 Génétique, Centre Hospitalier Universitaire, Saint-Etienne, France; 9 Médecine interne, Hôpital Saint Luc Saint Joseph, Lyon, France; 10 Génétique, Centre Hospitalier Universitaire, Tours, France; 11 Génétique, Centre Hospitalier Universitaire, Toulouse, France; 12 Service de génétique, Hôpital Femme Mère Enfant, Hospices Civils de Lyon, CRNL, CNRS UMR5292, INSERM U1028, Université Claude Bernard Lyon I, Lyon, France; 13 IGBMC, Hôpitaux Universitaires de Strasbourg, Université de Strasbourg, France; 14 Gillberg Neuropsychiatric Center, Gothenburg, Sweden; IGBMC/ICS, FRANCE

## Abstract

**Background:**

Intellectual Disability (ID) is characterized by deficits in intellectual functions such as reasoning, problem-solving, planning, abstract thinking, judgment, and learning. As new avenues are emerging for treatment of genetically determined ID (such as Down’s syndrome or Fragile X syndrome), it is necessary to identify objective reliable and sensitive outcome measures for use in clinical trials.

**Objective:**

We developed a novel visual analogical reasoning paradigm, inspired by the Progressive Raven’s Matrices, but appropriate for Intellectually Disabled patients. This new paradigm assesses reasoning and inhibition abilities in ID patients.

**Methods:**

We performed behavioural analyses for this task (with a reaction time and error rate analysis, Study 1) in 96 healthy controls (adults and typically developed children older than 4) and 41 genetically determined ID patients (Fragile X syndrome, Down syndrome and *ARX* mutated patients). In order to establish and quantify the cognitive strategies used to solve the task, we also performed an eye-tracking analysis (Study 2).

**Results:**

Down syndrome, *ARX* and Fragile X patients were significantly slower and made significantly more errors than chronological age-matched healthy controls. The effect of inhibition on error rate was greater than the matrix complexity effect in ID patients, opposite to findings in adult healthy controls. Interestingly, ID patients were more impaired by inhibition than mental age-matched healthy controls, but not by the matrix complexity. Eye-tracking analysis made it possible to identify the strategy used by the participants to solve the task. Adult healthy controls used a *matrix-based strategy*, whereas ID patients used a *response-based strategy*. Furthermore, etiologic-specific reasoning differences were evidenced between ID patients groups.

**Conclusion:**

We suggest that this paradigm, appropriate for ID patients and developmental populations as well as adult healthy controls, provides an objective and quantitative assessment of visual analogical reasoning and cognitive inhibition, enabling testing for the effect of pharmacological or behavioural intervention in these specific populations.

## Introduction

Intellectual Disability (ID) is characterized by a significant limitation in both intellectual functioning (which refers to general mental capacities such as reasoning, problem-solving, planning, abstract thinking, judgment, learning from experience) and in adaptive behavior as expressed in conceptual, social and practical adaptive skills (AAIDD, [1]). This disability originates before the age of 18. Impairment of reasoning abilities is a core symptom in ID patients [2]. In addition, inhibition (one of the three components of executive functions) represents a significant factor in practical and conceptual adaptive skills in children with ID [3, 4]. Several theoretical approaches have been developed to describe cognitive impairment in ID: (i) the *difference approach* which seeks to identify specific cognitive profiles with strengths and weaknesses, (ii) the *developmental approach* comparing ID patients with chronological (CA HC) and mental (MA HC) age-matched healthy control groups (developmental delay / deviance) and more recently (iii) the *neuroconstructivism approach* which focuses on developmental trajectories resulting from dynamic multidirectional interactions between genes, brain, cognition and environment [5–7].

Genetic causes of ID include visible chromosomal anomalies (such as Down’s syndrome (DS) [8] which is the most frequent aneuploidy), chromosomal micro deletion and monogenic diseases (such as mutation of the *ARX* gene [9–11] or mutation of the *FMR1* gene leading to Fragile X syndrome (FraX) [12]). DS and FraX are both well characterized syndromes including carefully detailed cognitive profiles, whereas *ARX* gene mutated patients (*ARX*) were more recently described [9–11]. The cognitive profile in DS is characterized by impairments in morphosyntax, verbal short-term memory, and explicit long-term memory; whereas visuospatial abilities, visuospatial short-term memory, and implicit long-term memory functions are relatively preserved [13–17]. The cognitive phenotype in FraX associates a relative strength in vocabulary capacity, visuo-perceptual abilities, with a weakness in verbal short-term memory, visuo-constructive abilities, visuo-spatial memory, linguistic processing, attention and executive functions [18–22].

Until very recently, treatments for ID have mainly focused on symptom management (including attention deficits and anxiety), and on minimizing complications related to comorbidities (e.g. epilepsy). New pharmacological treatment options that target the underlying defect related to each genetic mutation are currently being explored and may offer future opportunities to directly improve abstract reasoning [23]. The identification of reliable and sensitive outcome measures for use in clinical trials in Fragile X syndrome was recognized as a priority in the recent meeting convened by the National Institutes of Health [24]. Many therapeutic trials in ID patients use questionnaires completed by parents or caregivers, which rate the abnormal behaviors of patients [25]. Unfortunately, these tests are subjective, dependent on the judgment of the parents, and foregoing direct evaluation of the ID patients. In studies conducted by pharmaceutical companies, these questionnaires require selecting very impaired patients with pervasive behavioral problems in order to quantify improvement with the drug in question. This is a serious limitation if the effect of a new drug to improve specific cognitive abilities such as the use of more efficient reasoning strategies can be expected in less impaired patients.

In addition, most of the cognitive tests were developed to distinguish typically developing persons and ID patients, leading to a floor effect in the latter who systematically fail these tests. Therefore, these tests are not adapted to capture the potential effect of a drug within ID patients group.

To address the lack of a sensitive, reliable and simple outcome measure to assess non-verbal reasoning abilities (i.e. the core symptom in ID), we developed a novel visual analogical reasoning paradigm appropriate for ID patients, which was inspired by the Progressive Raven’s Matrices (PM). The PM test is very well correlated to the Intelligence Quotient. It is a non-verbal multiple choice measure of the reasoning component of Spearman's g factor, which is often referred to as general intelligence. In each test item, the subject is asked to identify the missing element that completes a pattern comprised of a 3x3 matrix. Several explanatory models have been proposed for analyzing cognitive abilities required in the PM test. Carpenter et al. [26] suggested that subject’s performances depended primarily upon their capacity to induce abstract relations and their working memory capacity. Other models evoked the speed of information processing [27] or the speed for hypothesis generation [28]. Finally, Bethell-Fox [29] introduced the idea that individuals may differ qualitatively because they use different strategies to solve the same task. Using eye-tracking analysis in healthy controls, Vigneau et al. were able to differentiate a “constructive matching strategy” *(matrix-based strategy)* involving the construction of an idealized answer which is then compared to the response alternatives of the response choice, from a “response elimination strategy” *(response-based strategy)* involving a process of feature comparison between elements of the problem and elements of the alternatives [29, 30]. Since ID patients uniformly fail on this Raven’s PM we developed a novel paradigm called “SimpleMatrices”. Furthermore, recent technological developments have enabled researchers to apply non-invasive eye-tracking technology to populations with ID [31] that enable directly testing the hypothesis that the deficit in ID performance is due to the use of inefficient strategies.

Before using this new task in ID patients, we validated this new paradigm in 96 healthy controls (adults and typically developed children older than 4). We then tested it in 41 genetically determined ID patients (FraX, DS and *ARX* patients). Two modalities were used to record the data: **behavioral data** (with a reaction time and error rate analysis, **Study 1**) and **eye-tracking data** (in order to establish and quantify the cognitive strategies used to solve the task, **Study 2**). For each group of patients, a comparison was performed with CA HC and MA HC. Thus, we were able to discriminate between developmental delay and specific syndrome related cognitive impairments. Mathematical modeling of the developmental trajectory for this task was also performed [7, 32].

In this report, we demonstrate that this novel task, “SimpleMatrices”, is appropriate for intellectually disabled patients. We show that objective and quantitative assessment of reasoning strategies can be performed in patients with moderate intellectual deficit. “SimpleMatrices” is a new tool that can be used as an outcome measure, enabling testing for a drug effect on reasoning in ID populations. Furthermore, specific deficits in reasoning abilities can also be identified between etiologically diverse ID patient groups.

## Materials and Methods

### Stimuli

As item difficulty is directly related to the number of response choices and the number of elements in an item [29], in our simplified paradigm matrix stimuli consist of four elements (instead of the nine cells in Raven’s), with two response choices (instead of the eight in Raven’s). Four parameters are involved in matrix creation: color (white, black or grey), form (round, square or triangle), number (one or two), and size (large or small). The number of relations between the items that need to be considered jointly to find the correct answer define the relational complexity of a matrix.

**Fig 1:**
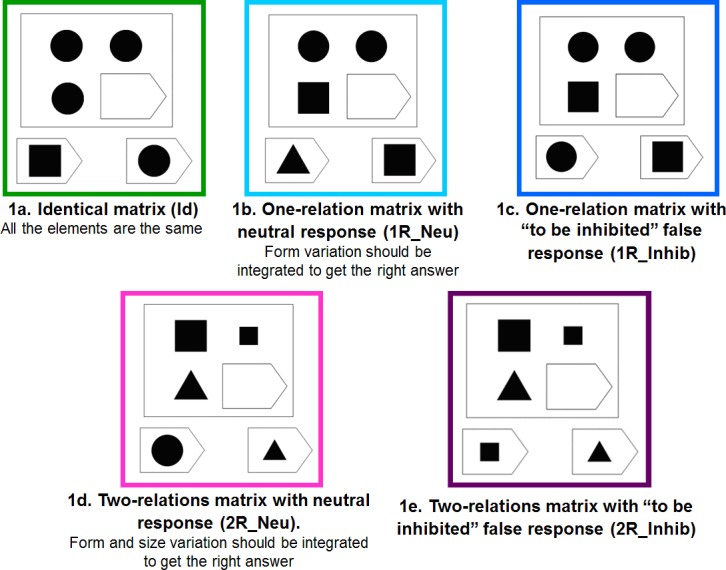
Example of the 5 different conditions included in the task (1a: Identical matrix (Id), 1b: One-relation matrix with neutral response (1R_Neu), 1c: One-relation matrix with “to be inhibited” false response (1R_Inhib), Two-relation matrix with neutral response (2R_Neu), Two-relation matrix with “to be inhibited” false response (2R_Inhib)).

There are **three levels of complexity**:

- **identical** (Fig 1A): the three elements of the matrix are the same;
- **“one-relation”** (Fig 1B): a very simple reasoning that requires consideration of only one varying parameter;
- **“two-relations”** (Fig 1D): variation in two parameters must be integrated to get the correct answer.

Furthermore, false-responses of **two different** types exist: a “**neutral**” response, which is a choice different from the items displayed in the matrix (Fig 1B and 1D) and a “**to be inhibited**” response, which is identical to one of the matrix items displayed (Fig 1C and 1E).

Thus, this task combines **5 different conditions**: identical matrices (Id), one-relation matrices with neutral responses (1R_Neu), one-relation matrices with to be inhibited responses (1R_Inhib), two-relations matrices with neutral responses (2R_Neu) and two-relations matrices with to be inhibited responses (2R_Inhib).

### Task Description

The participants were asked to identify the missing element that completes a pattern (Fig 1). The stimuli were displayed on a computer screen. Participants were told to find the best answer to fill in the missing “piece”. For the patients, immediate visual feedback via a happy or sad emoticon (in case of right or wrong answer, respectively) was given during the training to make the task easier to understand.

The paradigm consisted of four runs. Each matrix was preceded by a fixation cross. The subject then had to select the correct answer by pushing a button. The number of each type of condition was counterbalanced on each run, producing the same number of right and left answers. For each type of matrix, the type of variable relation (form, color, size, number) was also counterbalanced. The order of different conditions in the matrix display was randomized, as was the side of the correct answer. Each run consisted of 45 trials in the behavioral task and 15 trials in the eye-tracking task.

### Procedure

Recruitment was accomplished through the primary care doctor of the patients or the specialist who asked for the molecular or cytogenetic testing. This study was approved by the Ethical Committee of French Public Hospitals (Comité de Protection des Personnes Lyon-Sud Est II, 2010-A00327-32, 09/22/2010). After we informed the patients and their parents or guardians about the aims of the study, all of them gave written informed consent before the study procedure started. Child healthy controls were recruited from kindergartens, elementary and middle schools in Lyon. The parents of each child included in the study signed an informed consent before the study procedure started. The data were acquired in the National Reference Center for Rare Causes of Intellectual Disability (L2C2, CNRS UMR 5304, Lyon, France).

### Study 1: Behavioral Analysis

**Participants (details in Table 1)**.

**Table 1:** Characteristics of the patients and healthy controls included in Study 1 and 2.

	Adult HC	Child HC	DS patients	ARX patients	FraX patients
***Study 1*: *Behavioral analysis***					
Number of participants	n = 34	n = 62	n = 14	n = 13	n = 14
Chronological age : mean	27.7 years	[4–5 years] : n = 12	25.3 years	22.2 years	22.2 years
[range]	[18–43.7]	[6–7 years] : n = 11	[15.6–37.4]	[13.2–40.2]	[14.5–31.5]
		[8–9 years] : n = 11			
		[10–11 years]: n = 12			
		[12–13 years] : n = 9			
		[14–17 years] : n = 7			
Sex ratio (% males)	50%	45%	57%	100%	100%
Verbal IQ (VIQ) ^1^	-	-	49.1	50.2	52.0
			(SD 4.2)	(SD 4.8)	(SD 3.2)
Performance IQ (PIQ) ^1^	-	-	51.9	50.7	48.6
			(SD 5.3)	(SD 5.9)	(SD 8.8)
Total IQ (TIQ)^1^	-	-	47.9	47.3	49.7
			(SD 4.8)	(SD 8)	(SD 5.4)
Vocabulary Age^2^	-	-	10.1 years	8.0 years	10.7 years
			(SD 6)	(SD 1.6)	(2.6)
Mental Age^3^	-	-	7.4 years	7.1 years	5.8 years
			[5.75–9.5]	[4.25–11]	[4.25–7.5]
***Study 2*: *Eye-tracking analysis***					
Number of participants	n = 21	n = 38	n = 13	-	n = 14
Chronological age : mean,	27.6 years	[4–7 years] : n = 11	24 years	-	23.8 years
[range]	[18.3–41.8]	[8–11 years] : n = 15	[14.2–38.7]		[13.4–31.6]
		[12–16 years] : n = 12			
Sex ratio (% males)	71%	60%	62%	100%	100%

HC: Healthy Controls; DS: Down’s Syndrome; FraX: Fragile X syndrome; SD: Standard Deviation.

^1^Wechsler scales (the WAIS III in patients older than 16, the WISC IV in younger patients) were used for all patients but 6 Fragile X patients, for whom the Stanford Binet IV was used.

^2^Peabody Picture Vocabulary Test Revised (PPVT-R)

^3^Raven’s Coloured Progressive Matrices

- **Healthy controls** **(n = 96): 34 adult healthy controls** aged 18 to 45 years, without any history of neurological or psychiatric disorder and **62 children older than 4** were included in Study 1. None of them met exclusion criteria: history of neurological or psychiatric disorder, repetition of a grade, learning disability requiring rehabilitation (speech therapy, psychomotor or oculomotor therapy).
- **Intellectually Disabled patients** **(n = 41):** Three groups of ID patients were included in Study 1: **14 FraX patients** having a full mutation (more than 200 CGG repeats) of the *FMR1* gene located in Xq27.3; **14 DS patients** having a whole 21st chromosome trisomy on karyotypic analysis; **13 *ARX* mutated patients** having a mutation in the *ARX* gene (duplication of 24 bp (c.429-452 dup) in exon 2 second repeated PolyA tract, responsible for an insertion of 8 alanine residues in the ARX protein).

All ID patients had an IQ below 70 and the ability to perform the cognitive task. The 3 groups of ID patients did not differ significantly on Verbal IQ, Performance IQ, nor Total IQ. *ARX* and DS patients did not differ significantly on Raven’s PM. Nevertheless, since FraX patients had a significantly lower mental age on Raven’s PM than the other groups (p<0.05 and p<0.005 with *ARX* and DS patients respectively), specific mental age-matched groups were selected for each syndrome.

#### Data acquisition

The matrices were generated and displayed using Presentation software (http://www.neurobs.com). Each subject completed 4 runs. The task duration was around 15 minutes in healthy controls and 25 minutes in ID patients and young children. A break was allowed if necessary between two runs.

#### Data analysis

The raw data (logfiles from Presentation software) were analyzed automatically using Matlab 7.1. A Reaction Time (RT) and an Error Rate (ER) analysis were performed. The RT was defined as the time elapsing between the display of the matrix and the time when the subject pushed one of the two buttons to answer the problem. RT for the correct answers and ER were computed for each of the paradigm’s five conditions and for each subject included. Participants were excluded from the analysis if their ER for the two easiest conditions (‘Id’ and ‘1R_Neu’) was greater than the mean ER for all the 5 conditions from the group to which they belonged. This criterion was chosen to ensure that the task was well understood by all participants. This led us to exclude from the analysis: one DS patient, four *ARX* patients, three FraX patients, and one healthy child from the youngest group (4 to 5 years old).

#### Statistical analysis

Statistical analysis was performed using R software (http://www.r-project.org) on reaction time and error rate. The normality of the data distribution was first checked using the Shapiro and Wilk normality test. Then an ANOVA was applied in each group with two within group factors: **complexity of the matrix** (number of relations (0, 1, or 2) between items of the matrix needed to solve the problem) and **inhibition** (type of false response: either ‘neutral’ or ‘to be inhibited’). A significance level of p<0.05 was chosen. Post-hoc analysis was performed using the Scheffé test. The non-parametric Friedman test was applied when the data distribution was not normal. A between group analysis was also performed.

Each patient of each ID patient group (DS, *ARX* and FraX) was matched with both a CA HC subject and a MA HC subject. Group analyses were performed between each ID patient group and their respective chronological and mental age-matched control group.

Normative RT and ER data for this task were created in healthy controls and patients. Mathematical modelling of the developmental trajectory for this task in healthy controls was performed for RT and ER using “curvefit” toolbox in Matlab 7.1 (http://www.mathworks.fr).

Test-retest reliability (the degree to which test scores are consistent from one test administration to the next) was assessed within a session (between each of the four runs) in all healthy controls, DS and FraX patients using the Pearson correlation coefficient, while between session analysis was completed on the subset of participants who performed the task twice.

### Study 2: Eye-Tracking Analysis

#### Participants (see Table 1)

The same inclusion and exclusion criteria as used in Study 1 were applied respectively for each group.

- **Healthy controls (HC)****: (n = 59) 21 adult healthy controls** and **38 children older than 4** were included in Study 2. Ten out of the 21 adult healthy controls and 31 out of the 38 children were also included in Study 1.
- **Intellectually Disabled patients****: (n = 27)** Two groups of ID patients were included in Study 2: **14 FraX patients** (12 out of the 14 patients also included in Study 1) and **13 DS patients** (all also included in Study 1).

#### Data acquisition

Eye movements, fixations, and pupil diameters were acquired on the L2C2 CognitoScope platform (CNRS UMR5304) using a Tobii X120 eye-tracker, which is a remote binocular system with a temporal resolution of 60 Hz and a spatial resolution of 0.5 degree of visual angle. Thanks to the head movement compensation algorithms, no head restraints neede to be used. The eye-tracker, with an attached infrared light source to illuminate the pupil, was placed in front of the monitor where the stimuli were displayed. The device was below eye level, with an inclination of 19° relative to the horizontal and 70 cm from the participant. Each participant was seated on a comfortable and steady chair, with adjustable height to ensure good signal. Children were supplied footrests if needed. At the beginning of every eye-tracking session, we calibrated eye position for each participant. This calibration step was performed by having the participant look at colored dots in the center and four corners of the screen.

We used Tobii studio software to display the stimuli and record the eye-tracking data. In total, the analysis was performed on 12 matrices for each of the 5 conditions. During the task, an experimenter was present and controlled the quality of the eye-tracking data on a remote screen.

#### Data analysis

The eye-tracking data analysis was performed using Matlab 7.1 (http://www.mathworks.fr) (S1 File).

**Fig 2:**
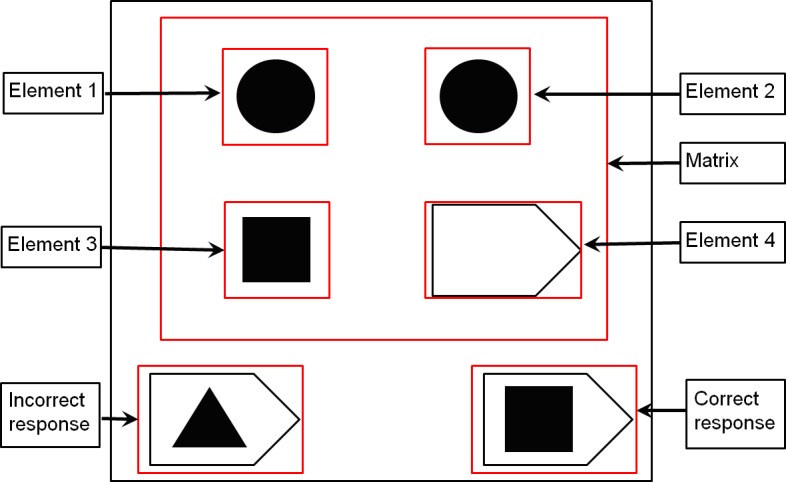
AOI (Area of Interest) defined for each stimuli of the eye-tracking task (the Matrix with its four elements and the two responses).

Three main Areas Of Interest (AOIs) were defined for each stimulus displayed: the Matrix and the two responses. Within the Matrix, four additional AOI were defined: element 1, element 2, element 3 and element 4 (Fig 2).

The following parameters were extracted and computed:

- **Number of transitions between the Matrix and the Responses** (defined as the number of toggles from the matrix area to the response areas or vice versa);
- **Latency of the first transition between the Matrix and the responses**;
- **Proportion of time on each AOI** (defined as the time spent on a given AOI divided by the time spent to solve the corresponding trial);
- **Latency of first fixation on each AOI** (defined as the time between the display of the stimulus on the screen and the first gaze fixation on each AOI).

The eye-tracking analysis was performed to characterize the cognitive strategy used to solve the reasoning task. If the participants used their peripheral vision from a fixed central gaze fixation, their eye-tracking data would not reflect the strategy they used. This led us to exclude from the analysis three adult healthy controls who spent 56.96%, 41.91% and 47.78% of the time respectively on element 4 (empty) for the identical condition.

#### Statistical analysis

Statistical analysis was performed using R software (http://www.r-project.org) on each of the parameters defined above. The normality of the data distribution was first checked using the Shapiro and Wilk normality test. Then an ANOVA was applied in each group with two within group factors: **complexity of the matrix** (number of relations (0, 1, or 2) between items of the matrix needed to solve the problem) and **inhibition** (type of false response: either neutral or to be inhibited). A significance level of p<0.05 was chosen. Post-hoc analysis was performed using the Scheffé test. The non-parametric Friedman test was applied if the data distribution was not normal. A between group analysis was also performed.

Each patient of each ID patient group (DS, and FraX) was matched with both a CA HC and a MA HC. Group analyses were performed between each ID patient group and their respective CA HC and MA HC groups.

## Results

### Study 1: Behavioral analysis

#### Results in Adult HC

**Fig 3:**
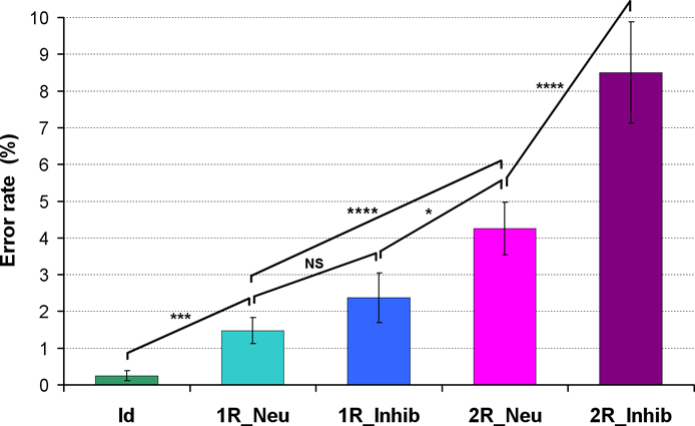
Result of the ER in 34 adult healthy controls (*:p<0.05, ***:p<0.005, ****:p<0.001, NS: Non Significant).

A significant **effect of the matrix complexity** was found on both RT and ER in adult HC (S1 Fig and Fig 3). They took significantly more time to solve the most complex matrices and made more errors on them. RT and ER were significantly greater for one relation compared to zero relation (p<0.001 and p<0.005 respectively) and greater for two relations compared to one (p<0.001 and p = 0.001 respectively).

There was no significant **effect of inhibition** on RT and ER for the easiest conditions (one relation) whereas a significant effect was observed for the most complex conditions (two relations) on both RT (p = 0.01, S1 Fig) and ER (p<0.001, Fig 3).

The interaction between matrix complexity (number of relations) and inhibition was statistically significant on both RT [*F*(1,33) = 20.48, p<0.001] and ER [*F*(1,33) = 8.18, p<0.01]: the effect of the matrix complexity was greater than the inhibition effect in adult HC.

#### Developmental trajectory in Typically Developed (TD) children

**Fig 4:**
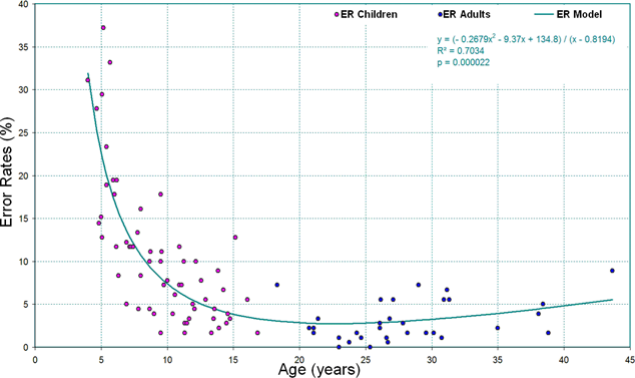
Non-linear regression analysis of ER with chronological age in healthy controls, children older than 4 and adults.

We performed a mathematical modeling of the developmental trajectory of RT and ER in healthy controls (older than 4) with the “curvefit” toolbox in Matlab 7.1 (http://www.mathworks.fr). The mathematical equations are indicated on S2 Fig and Fig 4. The coefficient of determination R^2^ and the significance of the correlation were 0.71 and p<0.001, respectively for RT and 0.70 and p<0.001 respectively for ER.

Normative data of the RT and ER for this task were created in healthy controls (both children and adults) and are reported in S1 and S2 Tables respectively.

In TD children (grouped in 2 year increments), a significant **effect of matrix complexity** was found on RT (S3 Fig). TD children took significantly more time to solve the most complex matrices: RT was significantly greater for one relation compared to zero relation (p<0.005 for each group) and greater for two relations compared to one (p<0.005 for each group).

A significant **effect of inhibition** was found on ER in TD children (S4 Fig). Moreover, the effect of inhibition on ER was significantly greater than the effect of matrix complexity (number of relations) until they were 6 years old. Interestingly, the ER was significantly greater for the 1R_Inhib condition than for the 2R_Neu condition, despite the higher complexity of the later condition. The significant effect of inhibition on ER for the easiest condition (one relation) was not observed in TD children >10 years old.

The lack of inhibition effect observed in the two youngest groups of TD children (4–5 and 6–7 years) on RT (S3 Fig) is associated with more impulsivity, leading to an apparent “opposite effect”, as the 4–5 year old children were significantly quicker for the items with ‘to be inhibited responses’ than with ‘neutral responses’. The 4–5 year old children seemed to be “attracted” by the wrong answer that looked like the matrix with a high ER. TD children older than 10 years old had a pattern for the RT very similar to that of the HC adults (S3 Fig).

#### Results in ID patients (DS, *ARX*, FraX): comparison between ID patients and control groups

**Fig 5:**
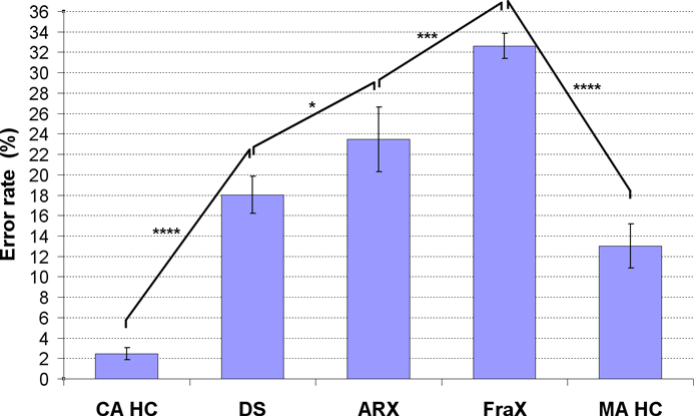
Group effect between DS, ARX, FraX, MA HC and CA HC on ER (MA HC: Mental Age-matched Healthy Controls, CA HC: Chronological Age-matched Healthy Controls, *:p<0.05, ***: p<0.005, ****: p<0.001).

A highly significant group effect was found on both RT and ER comparing ID patients to their respective CA HC: DS, *ARX* and FraX patients were significantly slower and made significantly more errors than their respective CA HC groups (S5 Fig and Fig 5, Table 2).

**Table 2:** Comparison between each group of patients and the respective CA HC and MA HC group for the behavioral data.

Group comparison	Age		RT	ER		
	Patients	Controls	Group effect	Group effect	[Matrix complexity*group] Interaction	[Inhibition*group] Interaction
**DS / CA HC**	25.3 years	25.4 years	p<0.001****	p<0.001****	p<0.001****	p<0.001****
	[15.6–37.4]	[14.6–38.1]				
**DS / MA HC**	7.4 years	7.3 years	NS	p<0.01**	NS	NS
	[5.75–9.5]	[5.4–9]				(p = 0.019* for 1R_Inhib)
**ARX / CA HC**	19.4 years	19.0 years	p<0.001****	p<0.001****	p<0.05*	p = 0.001**
	[13.2–39.1]	[13.6–38.8]				
**ARX / MA HC**	7.5 years	7.4 years	NS	p<0.001****	NS	p<0.05*
	[5.25–11]	[5–11.25]				
**FraX / CA HC**	23.5 years	23.1 years	p<0.001****	p<0.001****	p = 0.01*	p<0.001****
	[14.5–31.5]	[14.5–31.2]				
**FraX / MA HC**	5.7 years	5.9 years	NS	p<0.001****	NS	p<0.001****
	[4.25–7]	[4–7.2]				

CA HC: Chronological Age-matched Healthy Controls; MA HC: Mental Age-matched Healthy Controls; DS: Down’s Syndrome; FraX: Fragile X syndrome; ARX: ARX gene mutated patients; RT: Reaction Time; ER: Error Rate

*: p<0.05

**: p<0.01

****: p<0.001; NS: Non Significant.

We also performed a comparison between ID patients (DS, *ARX* and FraX patients) and their respective MA HC, allowing to distinguish what was related to a developmental delay in their performance and what could be considered as deviant.

When pooling all the conditions, no difference was observed on RT between ID patient groups and their respective MA HC (S5 Fig and Table 2). By contrast, ID patients made significantly more errors than their respective MA HC (Fig 5 and Table 2).

**Fig 6:**
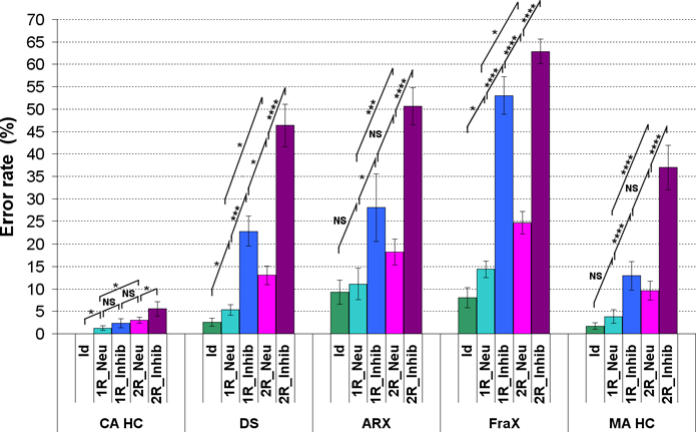
ER for each of the five conditions for each group of patients (DS, FraX, ARX) and for both CA HC and MA HC. (For display purposes and because the different chronological age-matched and mental age-matched control groups did not respectively differ significantly one from the other, the results of only one chronological age-matched control group and one mental age-matched control group are displayed).

A significant **effect of the matrix complexity** was found on both RT and ER in DS, *ARX* and FraX patients (S6 Fig and Fig 6), as well as in MA HC and CA HC. It took significantly more time and more errors were made for the most complex matrices. The interaction between the matrix complexity (number of relations) and the group on ER was not significant for each patient group when compared to their respective MA HC: all groups (each patient group and their respective MA HC group) were similarly impaired by the increase of matrix complexity (Table 2).

Unlike the CA HC, there was a significant **effect of inhibition** observed on ER even for the easiest conditions (one relation) in DS, *ARX* and FraX patients (Fig 6). The ER was significantly greater for the 1R_Inhib condition than for the 2R_Neu condition, despite the higher complexity of the later condition (Fig 6). The interaction between matrix complexity (number of relations) and inhibition was statistically significant for the ID patient groups: unlike the CA HC, the effect of inhibition on ER was greater than the matrix complexity effect.

#### Results in ID patients (DS, *ARX*, FraX): specific cognitive profile related to ID etiology

When pooling all the conditions, no difference was observed on RT between the different ID patients groups (mean RT of 2.84s for DS, 2.92s for FraX, 2.89s for *ARX;* S5 Fig and Table 2). But there was a significant group effect on ER between patients groups: FraX patients made significantly more errors than *ARX* patients, who made significantly more errors than DS patients (Fig 5).

The sensitivity to inhibition, although true for all patients groups, was especially striking for FraX patients who made significantly more errors than DS and *ARX* patients for the 1R_Inhib condition. The interaction between inhibition and group was significant on ER between both *ARX* and FraX patients and their respective MA HC: both groups (*ARX* and FraX patient groups and their respective MA HC groups) made more errors for the ‘to be inhibited false response’ conditions, but ARX and Fragile X patients were more impaired by the inhibition than their MA HC. The interaction between inhibition and group was not significant when comparing DS patients to MA HC. However, DS patients made significantly more errors than their MA HC in the ‘1R_Inhib’ condition (Table 2). Thus, DS patients did differ from the MA HC for the easiest condition with inhibition but not for the most complicated one.

#### Correlations with the neuropsychological tests

**Fig 7:**
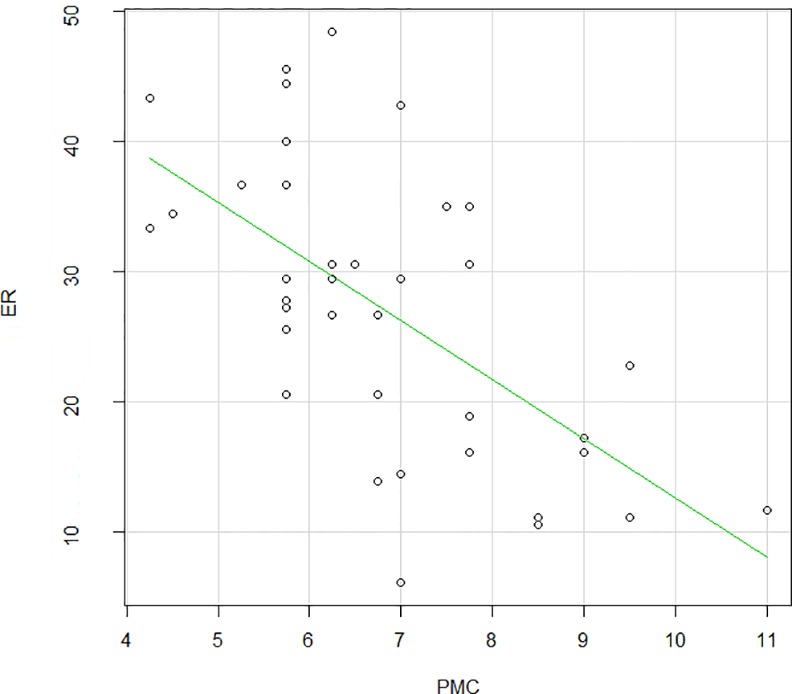
Correlation between ER and the Raven’s Coloured Progressive Matrices in ID patients (Pearson's product-moment correlation = -0.62, p<0.0001).

No correlation was found between RT and any of the cognitive measures (including Total IQ, Verbal and Performance IQ, Peabody Picture Vocabulary Test and Raven’s Coloured Progressive Matrices). But ER was strongly negatively correlated to the Raven’s Colored Progressive Matrices (Pearson's product-moment correlation = -0.62, p<0.001, Fig 7): the lower the ER was, the higher the mental age for the Raven’s.

#### Test-Retest reliability

**Fig 8:**
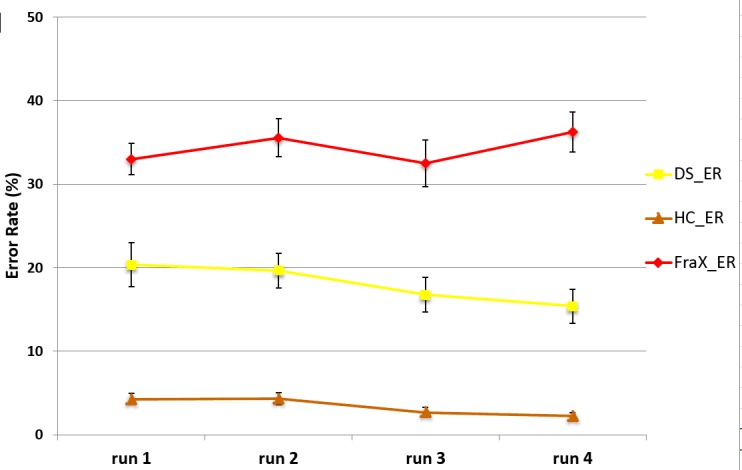
ER for each run in Adult healthy controls, DS and FraX patients.

Each participant performed four runs. We assessed the degree to which test scores were consistent from one test administration to the next (test-retest reliability) in adult healthy controls, DS and FraX patients. S7 Fig and Fig 8 displayed RT and ER for each of the three groups and for each run. There was no significant difference for any of the variables in the three groups (S8, S9 and S10 Figs). Pearson's product-moment correlation was computed between the 4 different runs for each group (S3 Table). These correlations, which were higher than 0.7 with p-values lower than 0.01, suggest good test-retest reliability.

Furthermore, we performed the same analysis of the ER and RT on half of the task (run 1 and 2). We found the same results as in the analysis presented above and using the four runs.

### Study 2: Eye-tracking analysis

#### Results in Adult HC

A significant **effect of the matrix complexity** (number of relations) was found on both the number of transitions (toggles) between the matrix and the responses and the latency of the first transition between the matrix and the responses in adult HC (S11 and S12 Figs). When the matrix became more difficult (increase in number of relations), adult HC made more transitions and spent more time on the matrix before switching to the responses, leading to a later occurrence of the first transition between the matrix and the responses.

There was no significant **effect of inhibition** on the number of transitions between the matrix and the responses in adult HC (S11 Fig).

Given that there were significant differences in RT between groups, we analyzed the proportional time on each AOI (defined as the time spent on a given AOI divided by the time spent to solve the corresponding trial) rather than the absolute time in order to allow the comparison of the different groups. In adult HC, the greatest **proportional time** was spent on the matrix. Moreover, they **first looked** at element 1, then at element 2, then at element 3 and then at the responses.

#### Results in TD children

There was a significant **age effect** on the number of transitions between the matrix and the responses. TD children older than 8 years were not statistically different from the adults, whereas children younger than 8 years made significantly more transitions. There was also a significant age effect on the latency of the first transition between the matrix and the responses: this latency increased as children got older. Children older than 12 years were not significantly different from adults.

A significant effect of the **matrix complexity** was found on the number of transitions between the matrix and the responses in TD children (S13 Fig). The number of transitions was significantly greater for the matrices with two than with one relation. The effect of the matrix complexity on the latency of the first transition between the matrix and the responses became significant only in children older than 12 years (S14 Fig).

Like for the adults, there was no significant **effect of inhibition** on the number of transitions between the matrix and the responses in TD children.

The greatest **proportion of time** was spent on the matrix in all groups of TD children. Adult HC spent significantly more time than any group of TD children on element 1, which reflects a full exploration of the matrix in order to deduce the correct answer.

Children 4 to 7 years old **first looked** at element 3, then at the responses, then at element 2, and then at element 1. Children 8 to 11 years old first looked at element 3, then at element 2, then at the responses, and then at element 1. Adolescents from 12 to 16 years old first looked at element 3, then at element 2, then at element 1, and then at the responses.

#### Results in ID patients: DS, ARX, FraX

There was a significant **group effect** on the number of transitions between the matrix and the responses: DS and FraX patients made more transitions than their respective CA HC (Table 3). The difference in number of transitions between the matrix and the responses between DS patients and FraX patients and their respective MA HC was not significant (Table 3).

**Table 3:** Comparison between each group of patients and the respective CA HC and MA HC group for the eye-tracking data.

Group comparison	Age		Number of Transitions Matrix/Responses		Latency of the first Transition Matrix/Responses		Proportion of observational time	
	Patients	Controls	Group effect	[Matrix complexity*group] Interaction	Group effect	[Matrix complexity*group] Interaction	Matrix	Responses
**DS / CA HC**	24 years	24.3 years	p<0.001****	NS	p<0.001****	p<0.001****	p<0.001****	p<0.005***
	[14.2–38.7]	[14.3–37.6]						
**DS / MA HC**	7.1 years	7.2 years	NS	NS	p<0.001****	NS	p<0.001****	p<0.05*
	[5.7–9.5]	[5.3–9.5]						
**FraX / CA HC**	23.8 years	23.2 years	p<0.001****	NS	p<0.001****	p<0.001****	p<0.001****	p<0.001****
	[13.4–31.6]	[12.3–31]						
**FraX / MA HC**	5.8 years	6.4 years	NS	NS	p<0.001****	NS	p<0.001****	p<0.001****
	[4.3–7.5]	[4.7–8.1]						

CA HC: Chronological Age-matched Healthy Controls; MA HC: Mental Age-matched Healthy Controls; DS: Down’s Syndrome; FraX: Fragile X syndrome

*: p<0.05

***: p<0.005

****: p<0.001; NS: Non Significant.

**Fig 9:**
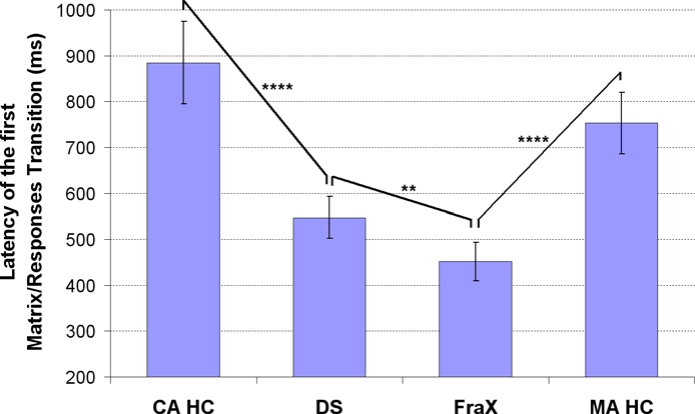
Analysis of the latency (ms) of the first transition between the matrix and the responses in DS, Fra X, and for both CA HC and MA HC. (For display purposes and because the different chronological age-matched and mental age-matched control groups did not respectively differ significantly one from the other, the results of only one chronological age-matched control group and one mental age-matched control group are displayed).

A significant group effect was also found on the latency of the first transition between the matrix and the responses: the first transition toward the responses occurred much earlier for the DS and FraX patients than for both CA HC and MA HC (Fig 9, Table 3). The healthy controls looked longer at the matrix before looking at the responses than the patients.

**Fig 10:**
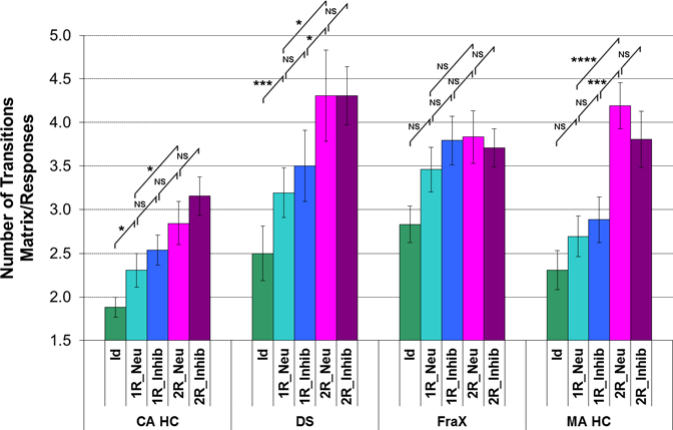
Analysis of the number of transitions between the matrix and the responses for the DS, FraX, and for both CA HC and MA HC. (For display purposes and because the different chronological age-matched and mental age-matched control groups did not respectively differ significantly one from the other, the results of only one chronological age-matched control group and one mental age-matched control group are displayed).

A significant effect of the **matrix complexity** (number of relations) on the number of transitions between the matrix and the responses was found in DS patients as well as in CA HC (Fig 10). Both groups made more transitions between the matrix and the responses for the conditions with two relations compared to the condition with one and for the conditions with one relation compared to the condition with zero. On the contrary, there was no significant effect of the matrix complexity on the number of transitions between the matrix and the responses in FraX patients.

**Fig 11:**
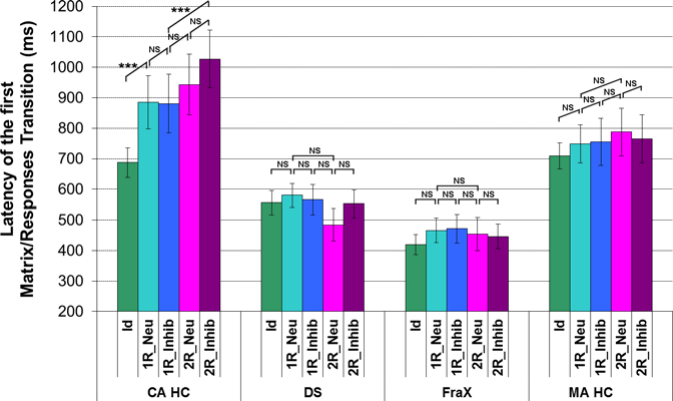
Analysis of the latency (ms) of the first transition between the matrix and the responses for each of the 5 conditions in DS, Fra X, and for both CA HC and MA HC. (For display purposes and because the different chronological age-matched and mental age-matched control groups did not respectively differ significantly one from the other, the results of only one chronological age-matched control group and one mental age-matched control group are displayed).

Unlike the CA HC, no effect of the matrix complexity was found on the latency of the first transition between the matrix and the responses in DS and in FraX patients: for all conditions, the latency of the first transition between the matrix and the responses was around 550ms and 400ms for the DS and FraX patients respectively (Fig 11).

**Fig 12:**
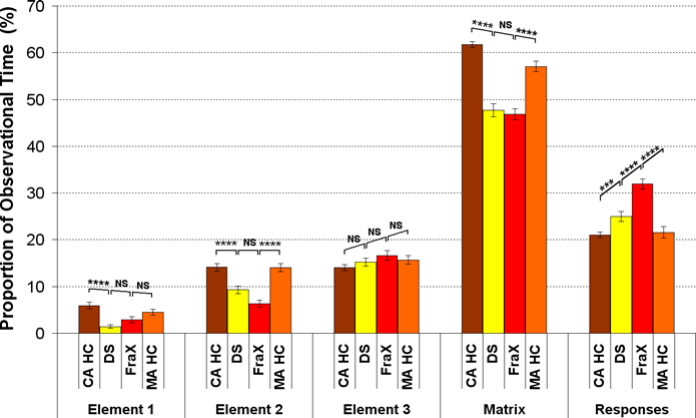
Analysis of the proportion of time on each AOI in DS, FraX, and for both CA HC and MA HC. (For display purposes and because the different chronological age-matched and mental age-matched control groups did not respectively differ significantly one from the other, the results of only one chronological age-matched control group and one mental age-matched control group are displayed).

A significant group effect on the **proportional time on each AOI** was found in DS and FraX patients compared to their respective CA HC and MA HC: DS and FraX patients spent significantly less time on the matrix (elements 1 and 2), and more on the responses than their respective CA HC and MA HC (Fig 12). DS and FraX patients were the two groups spending the least time on element 1.

The analysis of the latency of the first fixation on each AOI revealed a different **gaze pattern** for DS, FraX patients and CA HC. DS patients looked first at element 3, then at the responses, then at element 2 and finally at element 1. FraX patients looked first at the responses, then at element 3, then element 2 and finally at element 1. The analysis of the latency of the first fixation on each AOI revealed that DS patients and MA HC used the same gaze pattern looking at the AOI in the same order, even if controls looked significantly later on the responses.

## Discussion

We developed **a novel visual analogical reasoning paradigm**, inspired by the Progressive Raven’s Matrices, but **appropriate for ID patients**, enabling an objective and quantitative assessment of reasoning and inhibition abilities in this specific population.

**The behavioral analysis (Study 1)** showed **in adult HC** a significant effect of the matrix complexity (number of relations) on RT and ER, as well as an effect of inhibition but only for the most difficult conditions (two relations). They took more time to solve the matrix and made more errors with increasing matrix complexity and with the presence of inhibition. The effect of matrix complexity was much greater than the effect of inhibition in adult HC. Interestingly, we were able to quantify effects even in adult HC using this very simple paradigm. We did not observe any ceiling effect.

The **developmental data** showed that the effect of inhibition on ER was significantly greater than the effect of matrix complexity (number of relations) in TD children until they were 6 years old. The significant effect of inhibition on ER was not detected for the easiest condition (one relation) in TD children over the age of 10. This is in agreement with data from the literature about the time course of maturation of most executive functions [33–36]. Mathematical modeling of the developmental trajectory specific to this task (for RT and ER) was provided in HC [7, 32]. RT markedly decreased with age reaching a nadir at about 20 years of age in TD children then slowly increased again in adults. ER also strongly decreased with age. It would be interesting to include younger patients in order to determine whether this is also the case for ID patients.

**In ID patients**, as in adult HC, there was a significant effect of matrix complexity on RT and ER. But unlike in adult HC, the effect of inhibition was significant for the easiest conditions both on RT and ER in ID patients. They made more errors solving a simpler condition with inhibition than a more difficult condition without inhibition, showing that the effect of inhibition on ER was greater than the matrix complexity effect. **DS, *ARX* and FraX patients** were significantly slower and made significantly more errors than CA HC. ID patients made significantly more errors than their respective MA HC. ID patients and MA HC were similarly impaired when matrix complexity increased, but ID patients were more impaired than their respective MA HC by the inhibition. Executive inhibition deficit characterizes the three groups of ID patients we tested. The structure of executive functions with three components (working memory, inhibition and switching) seems to be similar in ID patients and healthy controls [3, 37].

**The comparison between the three patient groups** revealed that FraX patients made significantly more errors than *ARX* patients, who made significantly more errors than DS patients. FraX patients were very sensitive to inhibition even for the easiest conditions and made significantly more errors than DS and *ARX* patients for the easiest inhibition condition. Patients with FraX are known to exhibit attention deficit [18, 21, 22] and a deficit in executive functioning, especially in inhibition, working memory, cognitive flexibility and planning [20, 22, 38]. A broad impairment in executive functioning has also been suggested in DS patients, especially in cognitive flexibility, inhibition, working memory and planning [39]. Deficits in attention and executive functioning have both been found at a higher frequency in FraX patients in comparison with DS patients, especially inhibition deficits [21]. Our results are consistent with this finding. Very little is known about *ARX* patient neuropsychological profile. *ARX* patients seem to also have attention deficit [11]. It has been suggested in the literature that ID patients might have a specific profile of executive functioning with performance below their chronological age on all executive tasks; appropriate to their mental age in fluency or switching but below their mental age in inhibition, planning and non-verbal working memory [40, 41]. Our results suggest that this inhibition deficit is observed in ID patients, at a variable level according to the etiology of ID.

In order to analyze the cognitive strategies used to solve the task, an **eye-tracking data analysis** was also performed (**Study 2**). We adapted to our paradigm the parameters identified by Vigneau et al. to distinguish the cognitive strategies used to solve the reasoning task [30]. The eye-tracking analysis enabled us to differentiate a *Matrix-based strategy* involving the construction of an idealized answer which is then compared to the response alternatives of the response choice, from a *Response-based strategy* involving a process of feature comparison between elements of the problem and elements of the alternatives [29, 30], Table 4.

**Table 4:** Eye-tracking expected profiles for the matrix-based and the response-based strategies.

Expected profiles for the two distinct cognitive strategies		
	*Matrix-based strategy*	*Response-based strategy*
Number of transitions between the matrix and the responses	Few	Many
Proportion of time spent on the matrix	+++	+
Proportion of time spent on the responses	+	+++
Latency of the first transition between the matrix and the responses	increased	decreased
Latency of the first transition on the ‘responses’	increases with matrix complexity	No effect of matrix complexity

### In Adult HC, We Identified a *Matrix-Based Strategy*

Adult HC spent most of their time looking at the matrix. They started by looking at the element 1 (reflecting an evenly distributed examination of the matrix cells), and finished by looking at the responses. When the matrix complexity increased, adult HC spent more time on the matrix before switching to the responses, and made more alternations between the matrix and the responses. This is consistent with Vigneau’s finding that higher ability subjects typically spent more time at the encoding phase of the process, especially when the number of rules increases, and demonstrates that our very simple paradigm was sensitive enough to reproduce these effects.

### In DS and FraX Patients, We Identified a *Response-Based Strategy* (Table 4)

DS and FraX patients made more transitions than their respective CA HC. The first transition towards the responses occurred much earlier for DS and FraX patients than for their respective CA HC. The matrix complexity had no effect on the latency of the first transition between the matrix and the responses in DS and FraX patients. Moreover, DS and FraX patients spent significantly less time on the matrix, but more on the responses than CA HC. The gaze pattern in FraX patients was characterized by the fact that they first looked at the responses.

**The developmental data** showed a significant age effect on the number of transitions between the matrix and the responses, which decreases when children get older. There was also a significant age effect on the latency of the first transition between the matrix and the responses, which increases when children get older.

When DS and FraX patients were compared **to their respective MA HC group** MA HC looked longer at the matrix before switching to the responses. MA HC spent also proportionally more time on the matrix and less on the responses than both DS and FraX patients. The differences in gaze pattern between DS and FraX patients and their respective MA HC were striking. ID patients’ strategy is not only immature related to their delay, but also different from the typically developed children.

The eye-tracking analysis made it possible to identify the strategy used by participants to solve the task. ID patients are not able to explicitly explain the strategy they used to solve the task, but with eye-tracking analysis, we can understand how they performed the task, which is crucial information in order to help them improve their performance through remediation strategies.

It is interesting to note that the eye-tracking pattern allowed us to distinguish between DS and FraX: the first transition between matrix and responses occurred significantly later and FraX patients spent significantly more time on the responses than DS patients. Thus, the eye-tracking analysis of this new paradigm revealed not only differences between ID patients and controls, but also between the different etiologies of ID–a distinction that would not be possible using the Raven’s matrices [42].

### Limitations and Future Perspectives

As our goal was to quantitatively assess visual analogical reasoning abilities in ID patients, we designed the paradigm to be as simple as possible. However, this meant limiting to only two response choices, leading to a high probability of correct answers. Despite this limitation, we observed significant differences between groups. Furthermore, we only acquired eye-tracking data in two ID populations (DS and FraX). It would be interesting to study other ID etiologies in the future (such as *ARX*, *PQBP1* or Williams syndrome) to check if a specific eye-tracking pattern exists for each of them. Finally, we were able to determine the strategies that ID patients used to solve the task, but not the neural correlates associated with this cognitive strategy. Further study using fMRI would be needed in the future to determine these neural correlates.

We validated this novel visual analogical reasoning task, SimpleMatrices, in both ID patients and healthy controls. The test-retest reliability was assessed in patients and in healthy controls and showed high validity. We suggest that this paradigm, appropriate for ID patients and developmental populations, provides an objective and quantitative assessment of visual analogical reasoning and cognitive inhibition, and thus might be useful for the evaluation of pharmacological treatment or behavioural intervention in these specific populations.

## Supporting Information

S1 Fig Result of the Reaction Time in 34 adult healthy controls (*:p<0.05, ****:p<0.001, NS: Non Significant). (TIFF)

S2 Fig Non-linear regression analysis of RT with chronological age in healthy controls, children older than 4 and adults. (TIFF)

S3 Fig Result of the RT in 62 children older than 4, grouped in 2 years increment. (TIFF)

S4 Fig Result of the ER in 62 children older than 4, grouped per 2 years. (TIFF)

S5 Fig Group effect between DS, FraX, ARX, MA HC and CA HC on RT (MA HC: Mental Age-matched Healthy Controls, CA HC: Chronological Age-matched Healthy Controls, *:p<0.05, NS: Non significant). (TIFF)

S6 Fig RT for each of the five conditions for each group of patients (DS, FraX, ARX) and for both CA HC and MA HC. (For display purposes and because the different chronological age-matched and mental age-matched control groups did not respectively differ significantly one from the other, the results of only one chronological age-matched control group and one mental age-matched control group are displayed).
(TIFF)

S7 Fig RT for each run in Adult healthy controls, DS and FraX patients (TIFF)

S8 Fig RT for each run and each condition in Adult healthy controls. (TIFF)

S9 Fig RT for each run and each condition in DS patients. (TIFF)

S10 Fig RT for each run and each condition in FraX patients. (TIFF)

S11 Fig Analysis of the number of transitions between the matrix and the responses in adult healthy controls (*: p<0.05, **: p<0.01). (TIFF)

S12 Fig Analysis of the latency of the first transition between the matrix and the responses in adult healthy controls (*: p<0.05, ***: p<0.005, ****: p<0.001). (TIFF)

S13 Fig Analysis of the number of transitions between the matrix and the responses for the children older than 4 years and for each of the five conditions. (TIFF)

S14 Fig Analysis of the latency (ms) of the first transition between the matrix and the responses in children older than 4 years. (TIFF)

S1 File Eye-tracking analysis (methodology). (PDF)

S1 Table Normative data of the RT in healthy controls (both children and adults). (DOC)

S2 Table Normative data of the ER in healthy controls (both children and adults). (DOC)

S3 Table Pearson's product-moment correlation between the 4 different runs for each group. (DOC)

## Abbreviations

AOI: Area Of Interest
*ARX* gene: Aristaless Related Homeobox gene
*ARX* patient: *ARX* gene mutated patient
CA HC: Chronological Age-matched Healthy Control
DS: Down’s syndrome
ER: Error Rate
*FMR1* gene: *Fragile X Mental Retardation 1* gene
FraX: Fragile X syndrome
HC: Healthy Control
ID: Intellectual Disability
IQ: Intelligence Quotient
MA HC: Mental Age-matched Healthy Control
PM: Progressive Raven’s Matrices
RT: Reaction Time
TD: Typically Developed
