# Supplementary figures and images for: A Novel Analog Reasoning Paradigm: New Insights in Intellectually Disabled Patients
Source: PLoS One. 2016 Feb 26;11(2):e0149717. doi: 10.1371/journal.pone.0149717 (PMC4771701; doi:10.1371/journal.pone.0149717)

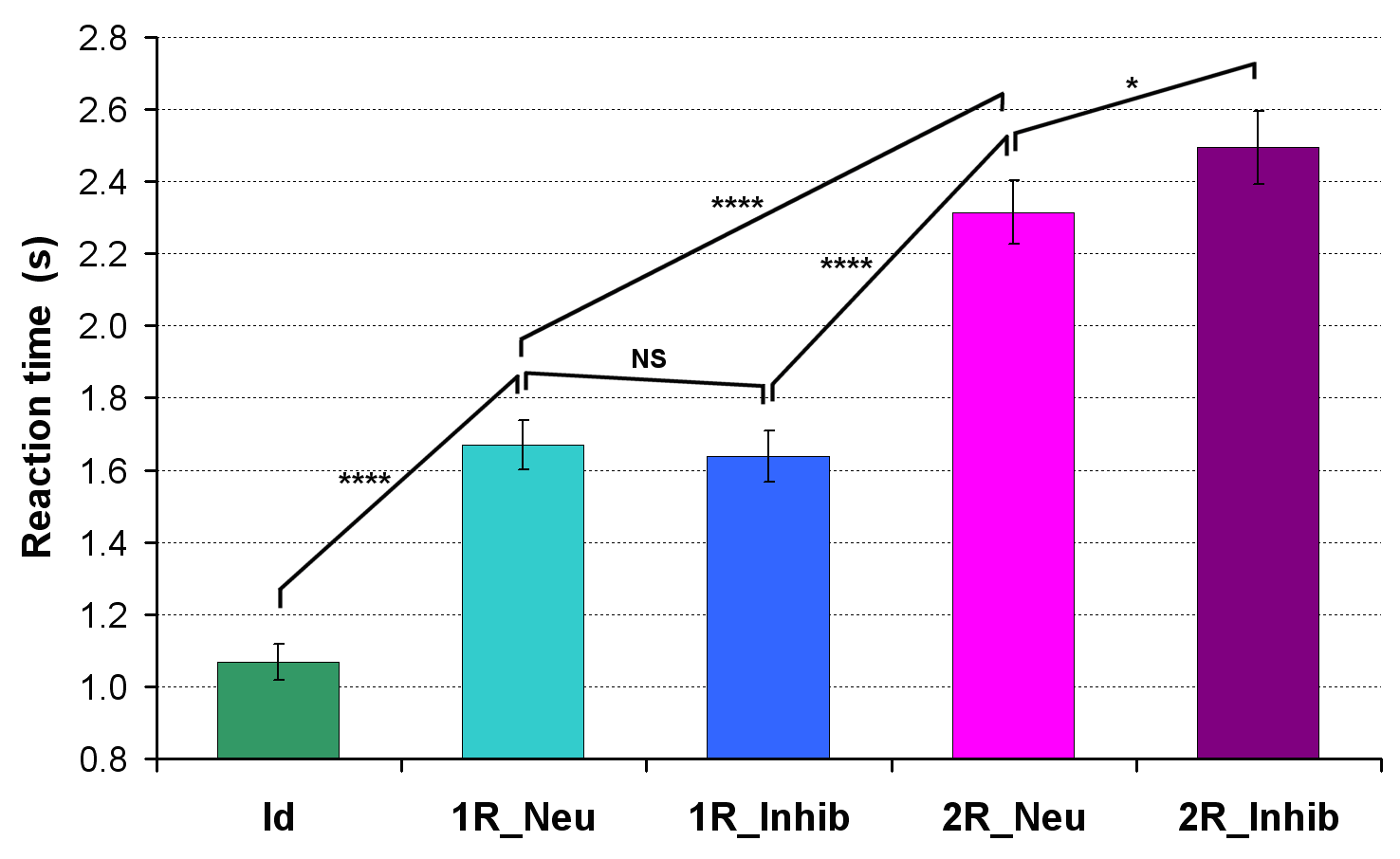

Supplement: S1 Fig — (TIFF) [file pone.0149717.s001.tiff]

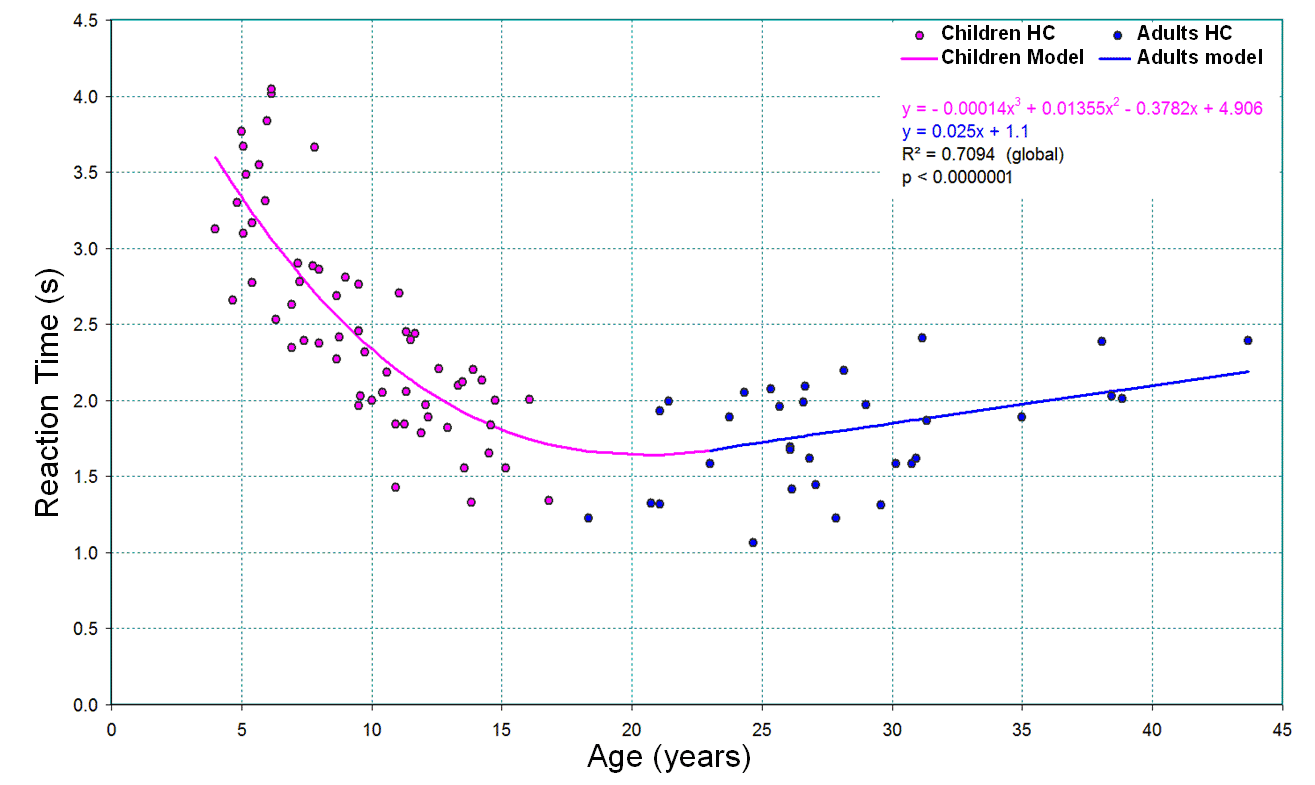

Supplement: S2 Fig — (TIFF) [file pone.0149717.s002.tiff]

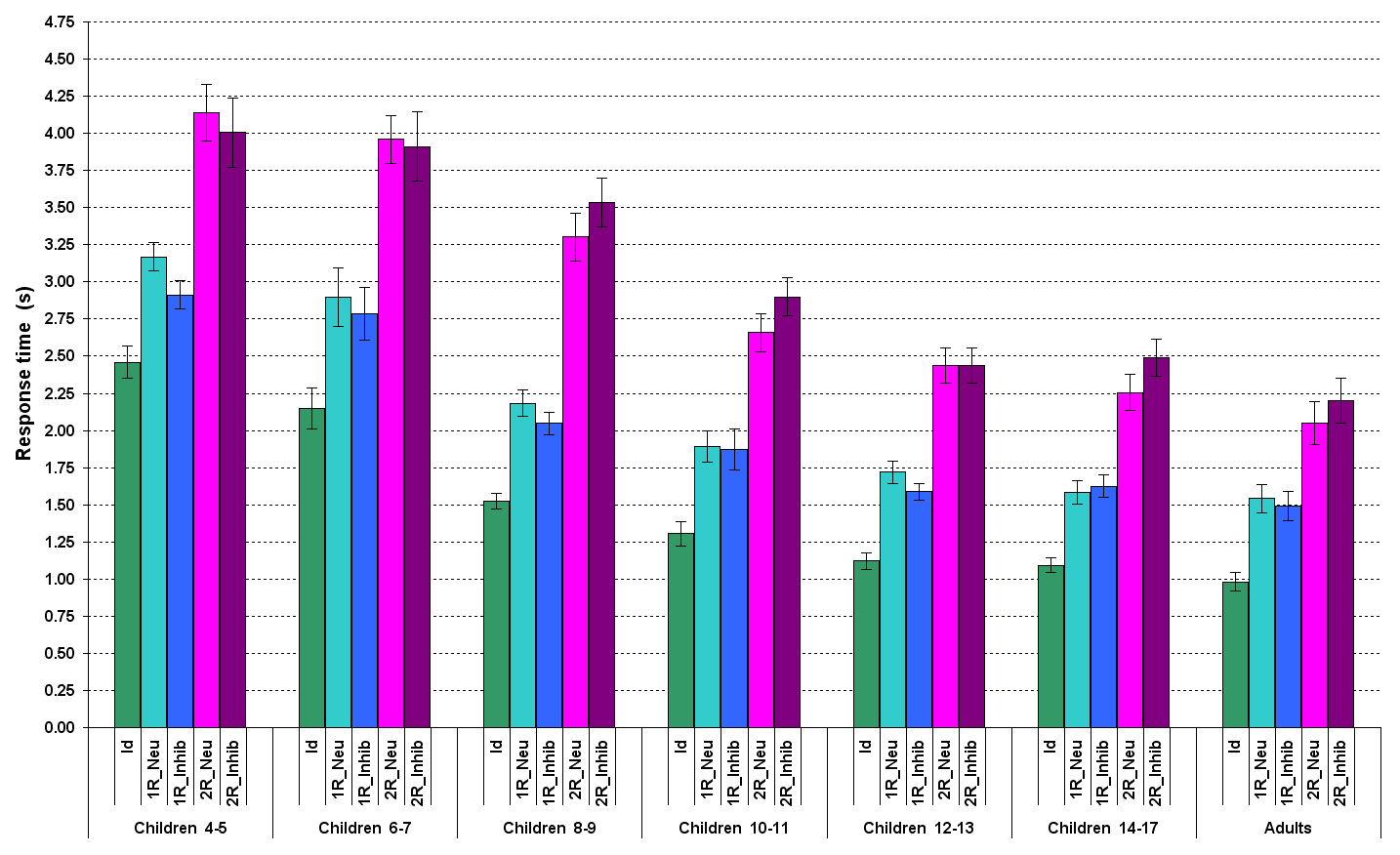

Supplement: S3 Fig — (TIFF) [file pone.0149717.s003.tiff]

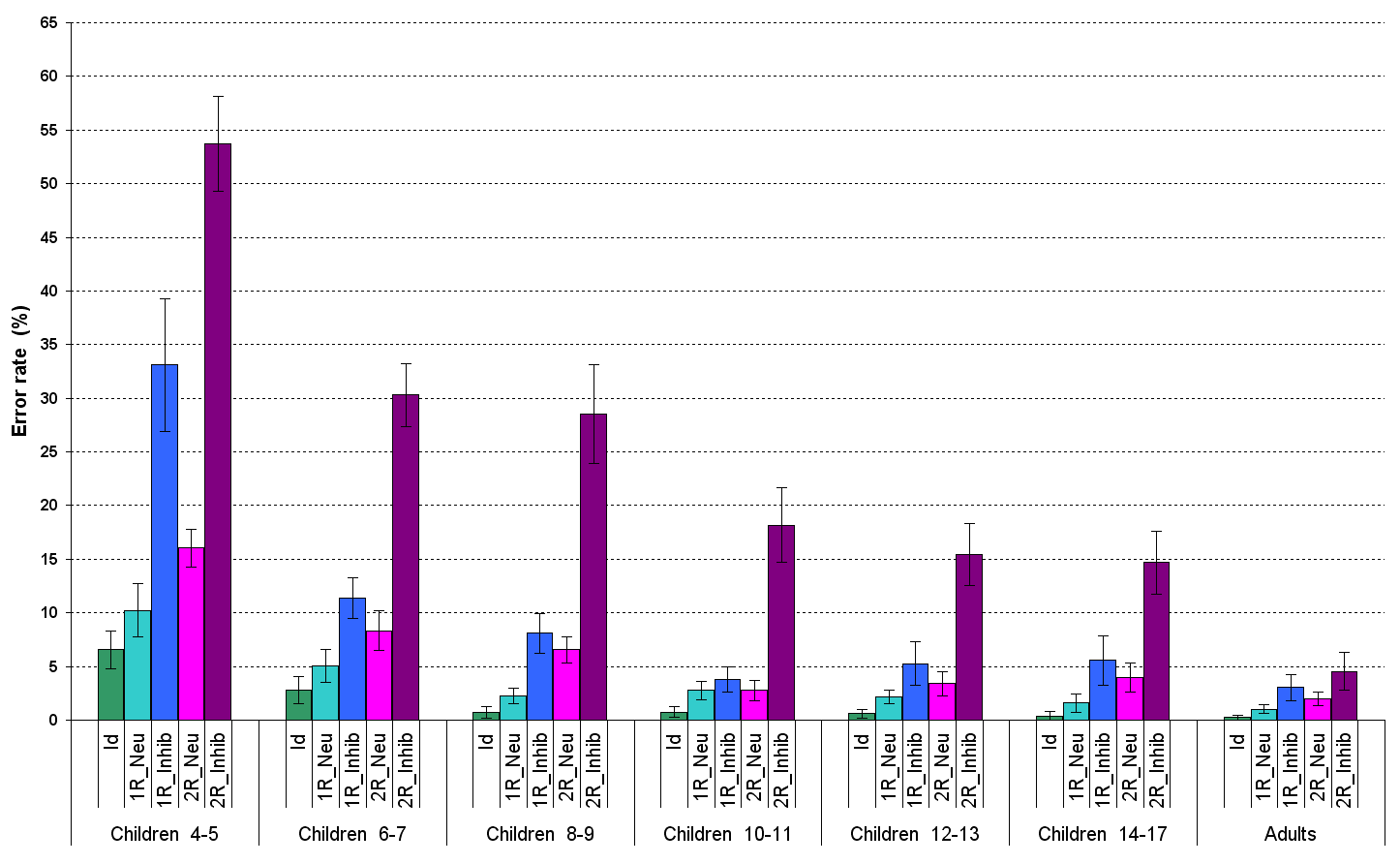

Supplement: S4 Fig — (TIFF) [file pone.0149717.s004.tiff]

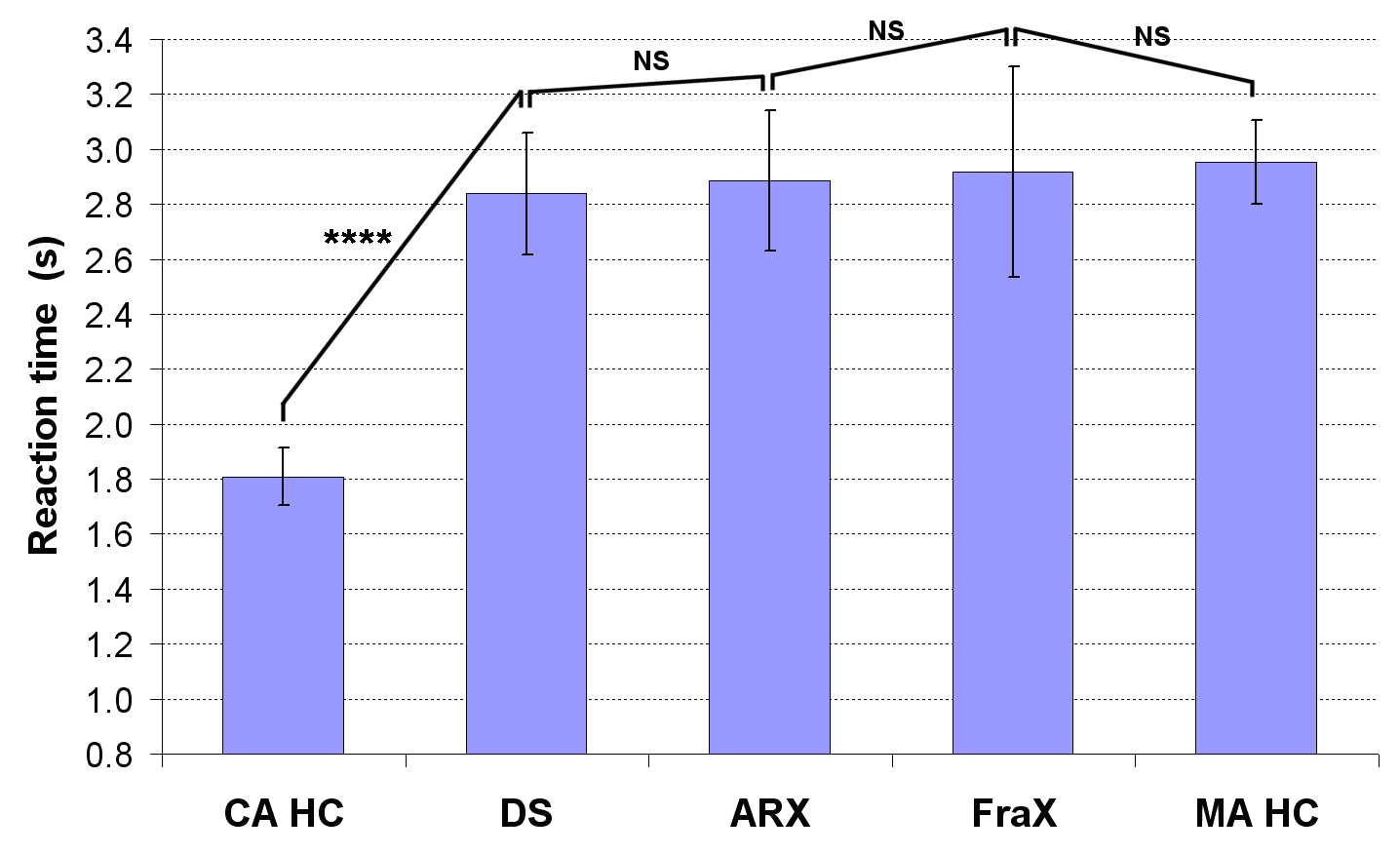

Supplement: S5 Fig — (TIFF) [file pone.0149717.s005.tiff]

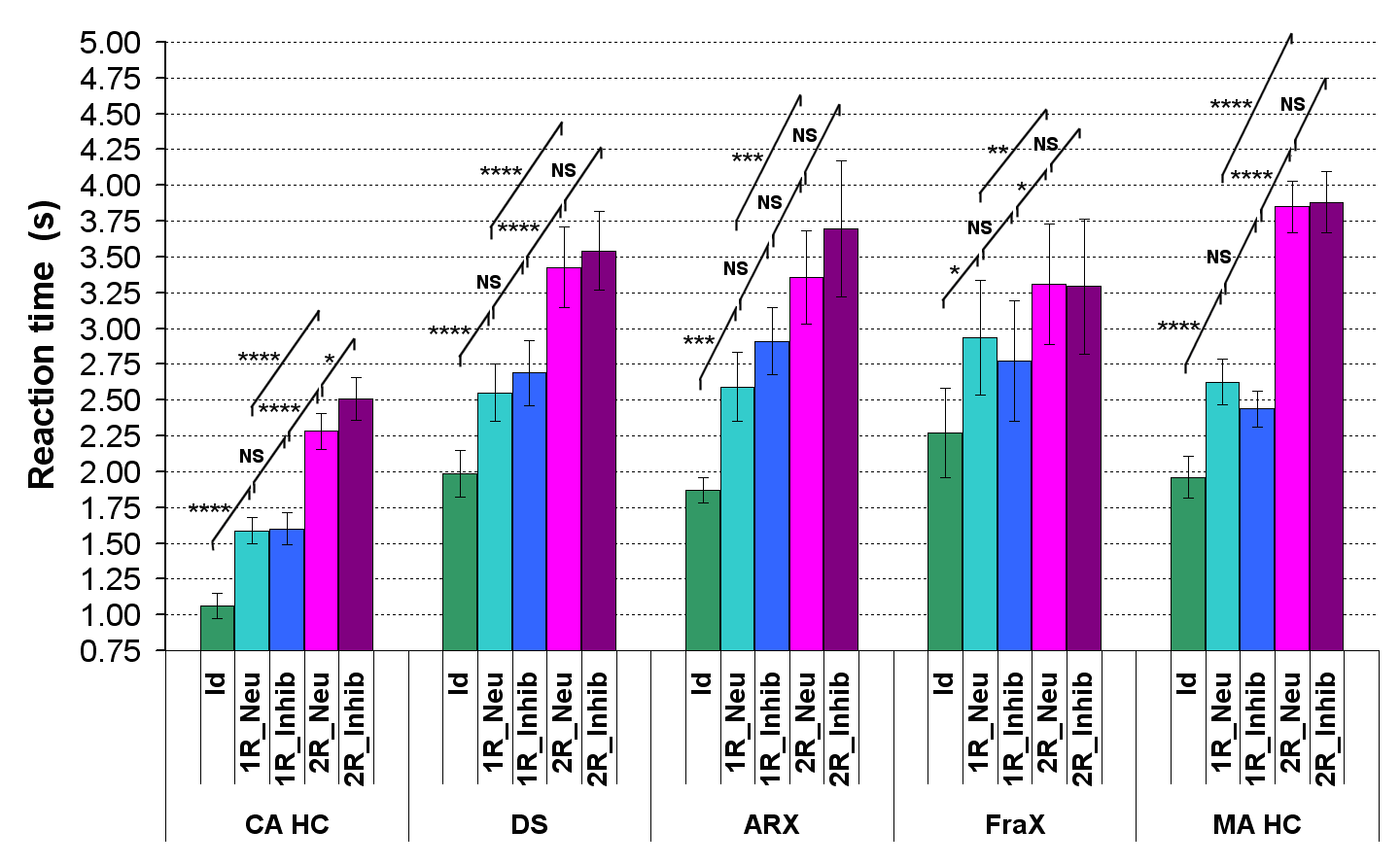

Supplement: S6 Fig — (For display purposes and because the different chronological age-matched and mental age-matched control groups did not respectively differ significantly one from the other, the results of only one chronological age-matched control group and one mental age-matched control group are displayed). (TIFF) [file pone.0149717.s006.tiff]

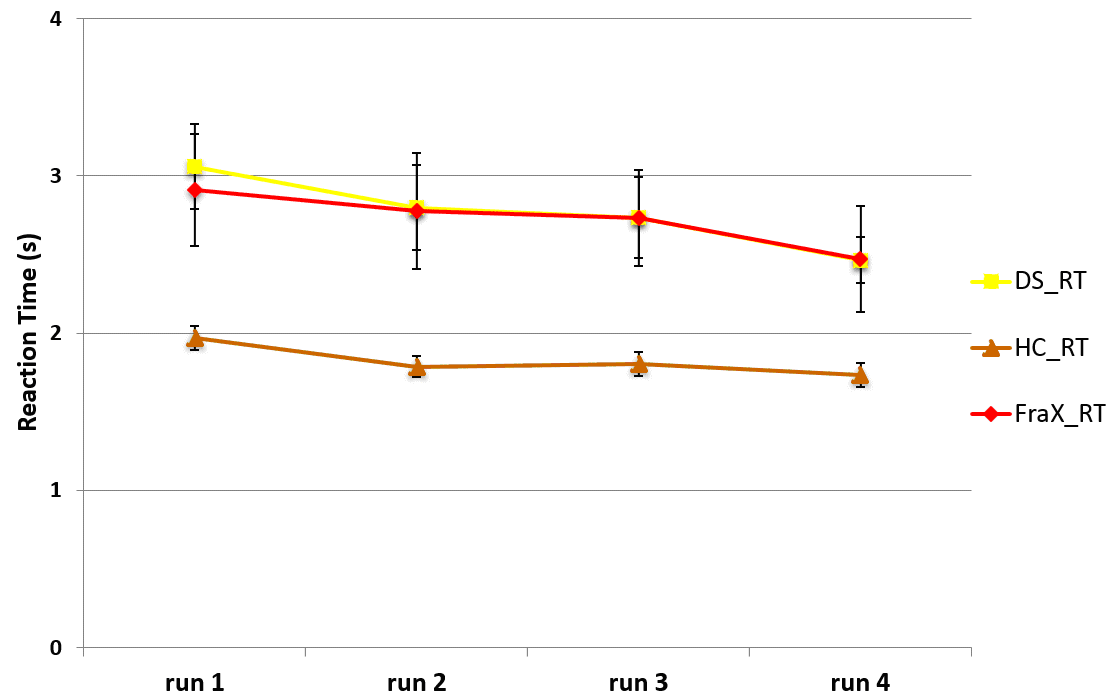

Supplement: S7 Fig — (TIFF) [file pone.0149717.s007.tiff]

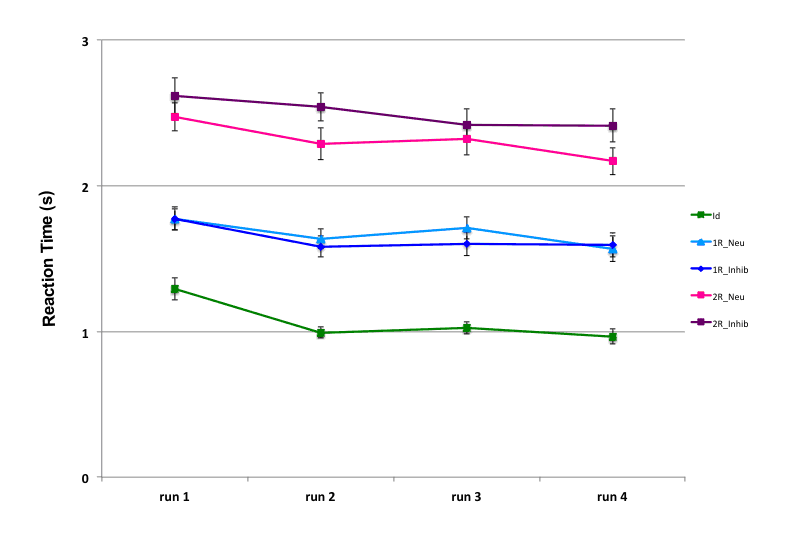

Supplement: S8 Fig — (TIFF) [file pone.0149717.s008.tiff]

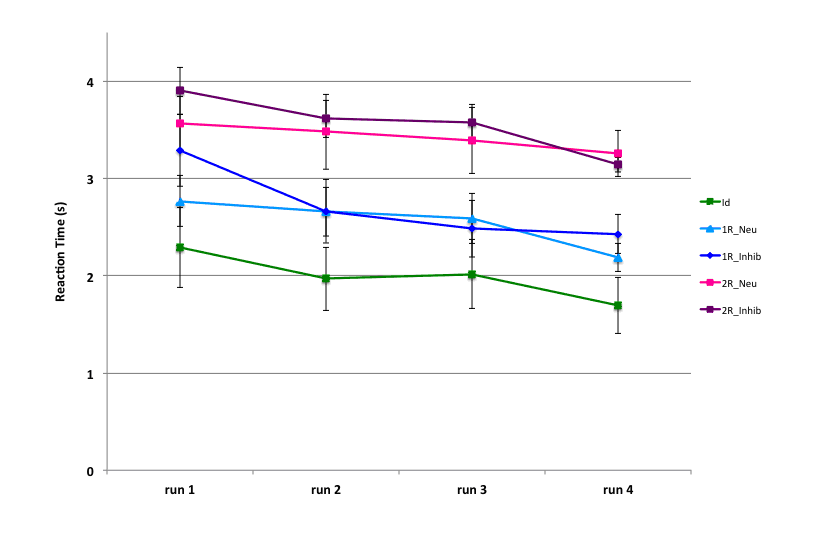

Supplement: S9 Fig — (TIFF) [file pone.0149717.s009.tiff]

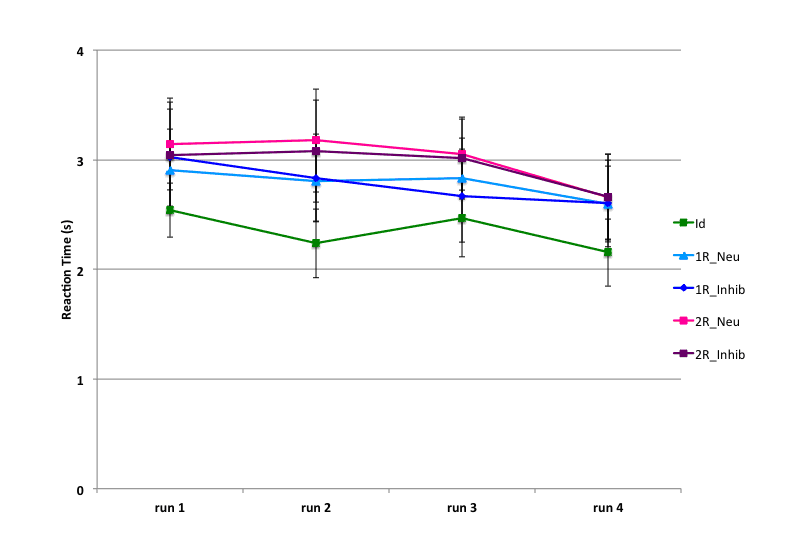

Supplement: S10 Fig — (TIFF) [file pone.0149717.s010.tiff]

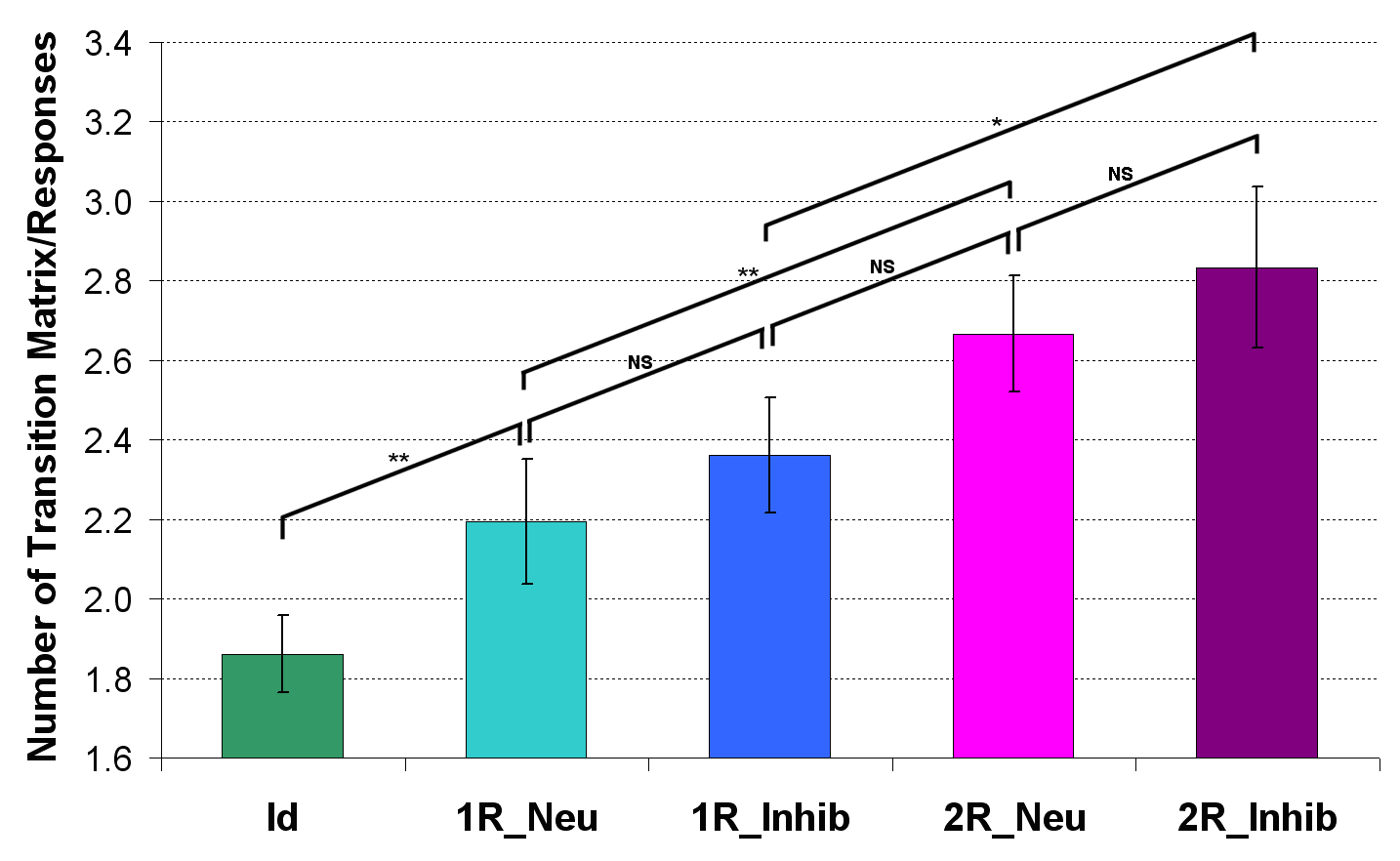

Supplement: S11 Fig — (TIFF) [file pone.0149717.s011.tiff]

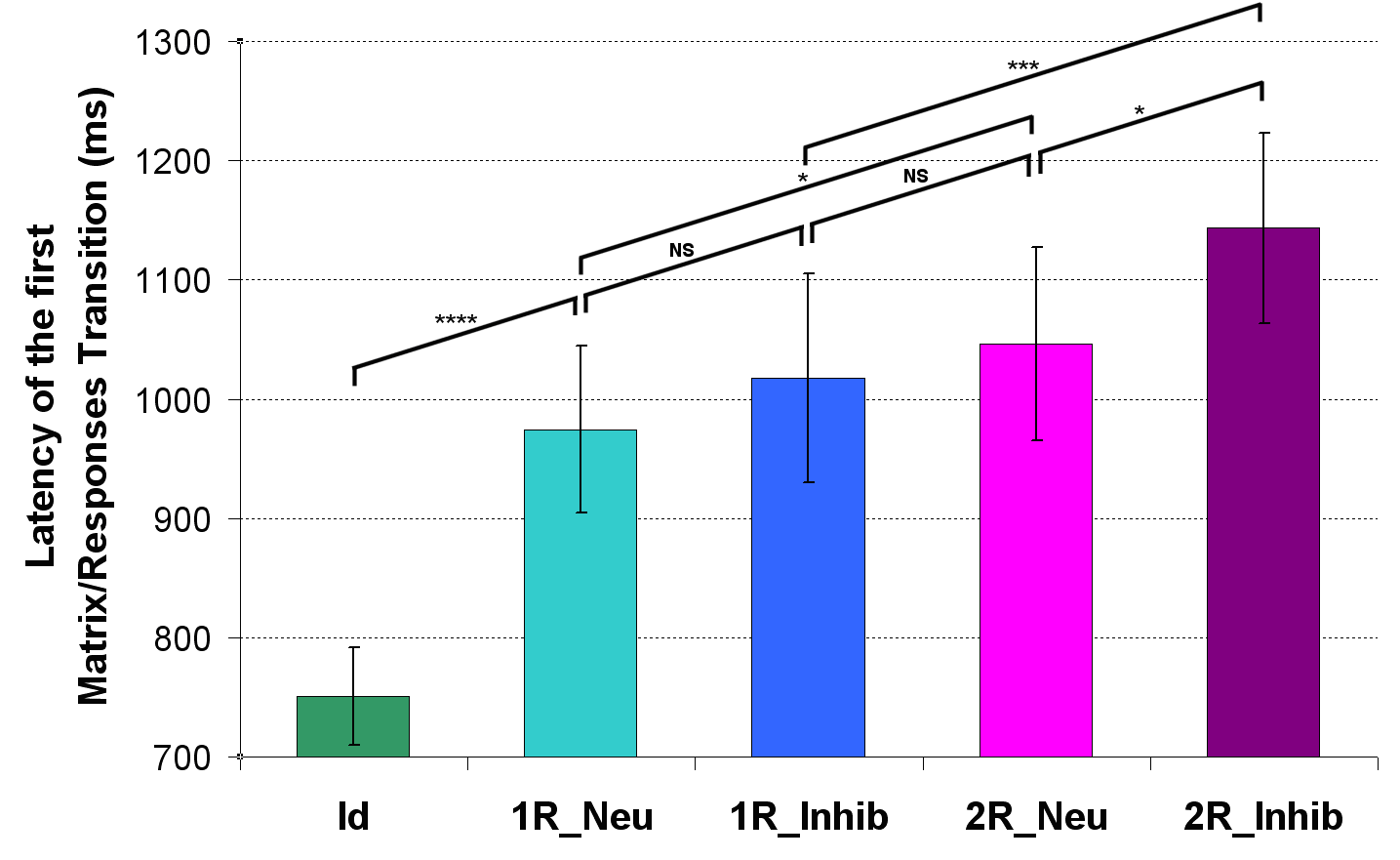

Supplement: S12 Fig — (TIFF) [file pone.0149717.s012.tiff]

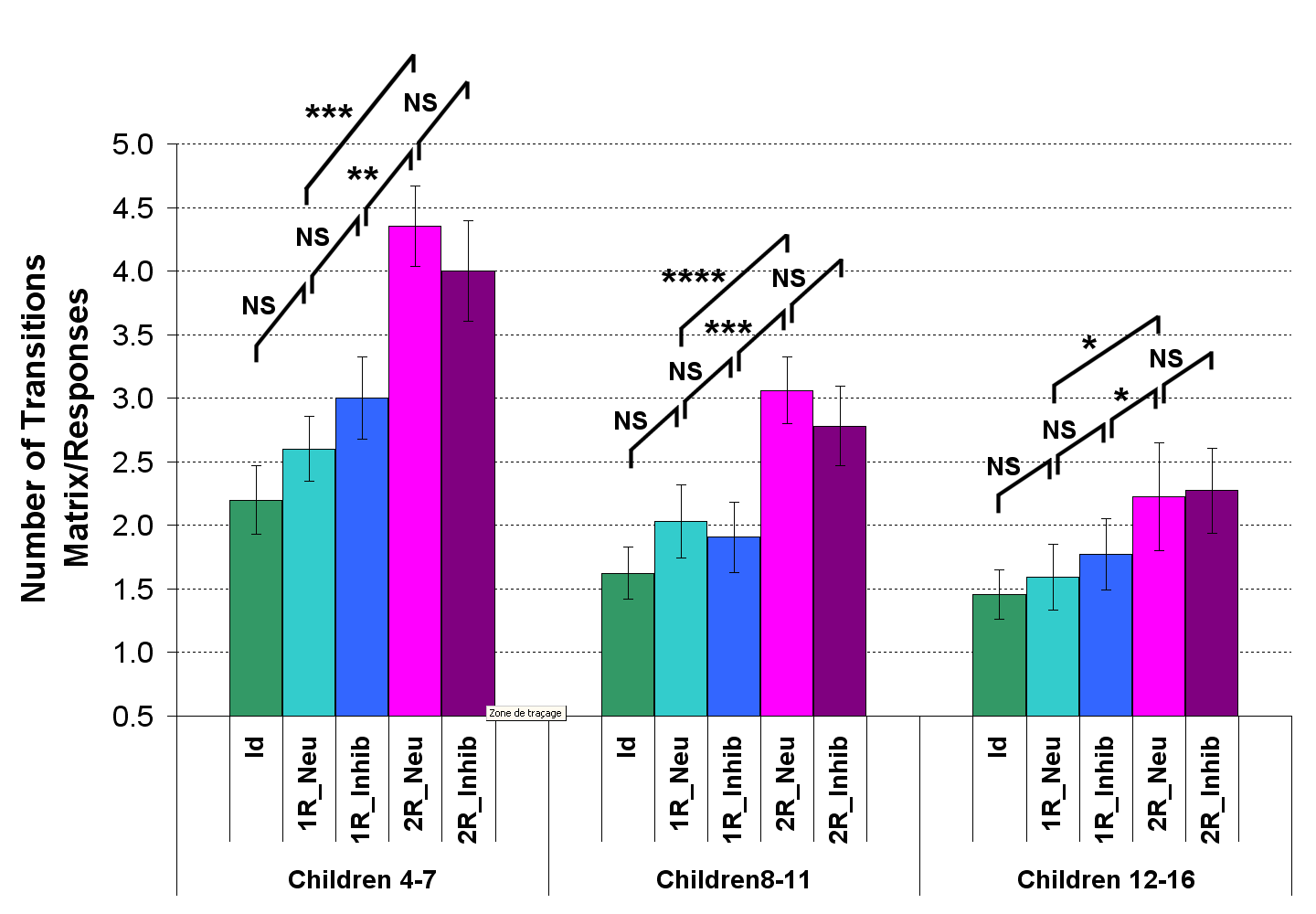

Supplement: S13 Fig — (TIFF) [file pone.0149717.s013.tiff]

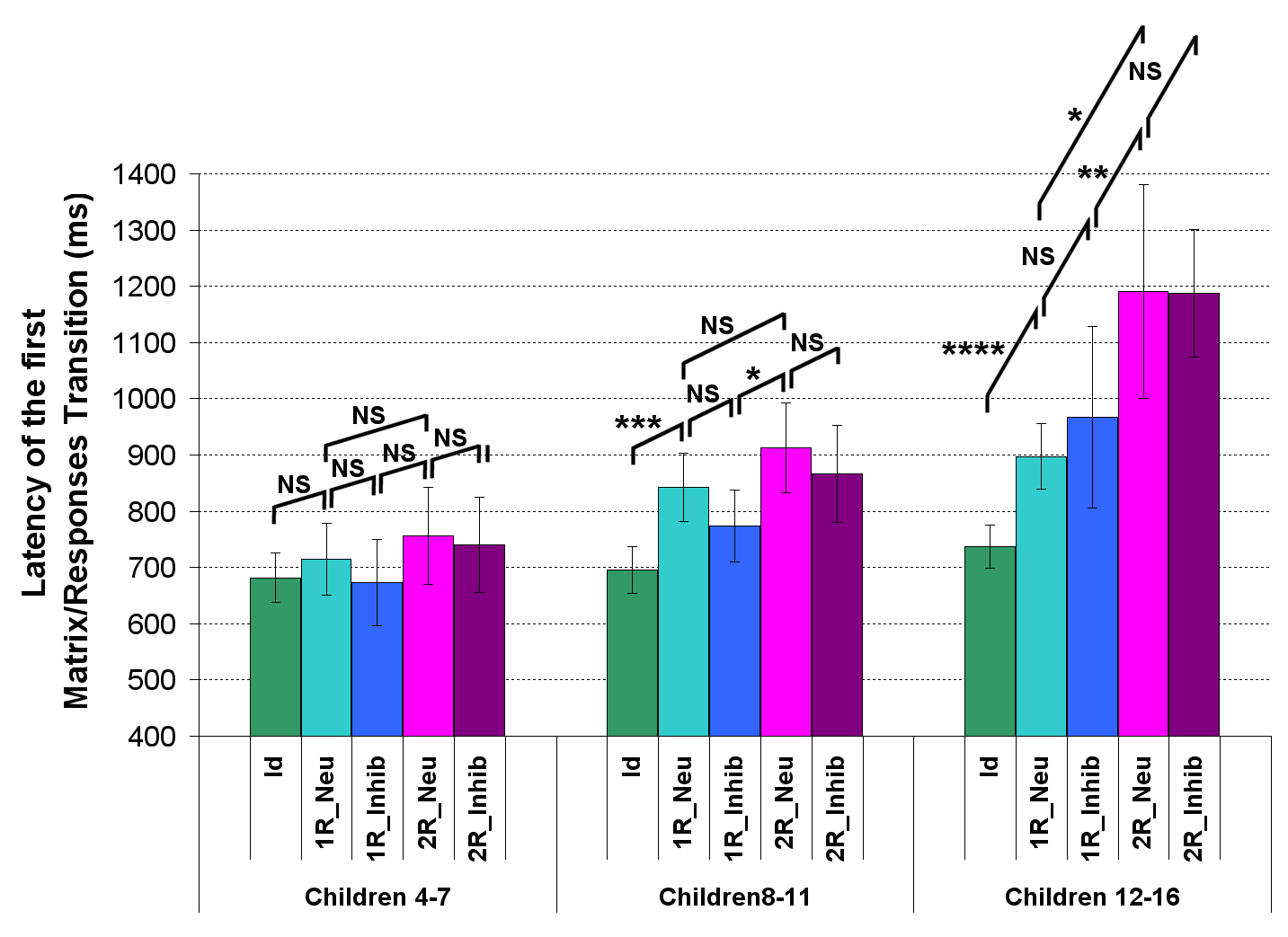

Supplement: S14 Fig — (TIFF) [file pone.0149717.s014.tiff]
