# Supplementary material for: A Novel Analog Reasoning Paradigm: New Insights in Intellectually Disabled Patients
Source: PLoS One. 2016 Feb 26;11(2):e0149717. doi: 10.1371/journal.pone.0149717 (PMC4771701; doi:10.1371/journal.pone.0149717)
Supplement: S1 File — (PDF) [file pone.0149717.s015.pdf]

We first developed our analysis using Tobii Studio filtering methods, but as the number of subjects increased we had to automate this procedure outside Tobii Studio to avoid repetitive, error prone manual manipulations. Furthermore, we were interested in analyzing the number of transitions from one Area of Interest (AOI) to another, which was not possible using Tobii Studio. We read the sqlite database from Tobii Studio using the mksqlite functions from Matlab developed by Martin Kortman (http://mksqlite.berlios.de). We first applied a filter (very similar to the one used in Tobii Studio) to differentiate saccades from fixations and interpolate missing data occurring during blinks. Given that the data were acquired with a temporal resolution of 60 Hz, we labelled every sample as either missing data, a saccade or a fixation. When data were missing for less than 400 ms, we defined this as a physiological blink [17]. A saccade is defined when the distance between two successive data samples exceeds a threshold h. To take into account the noise inherent to the raw data, we applied a moving average filter (M_{avg}) of r order before computing the distance between the samples, as it is performed in Tobii Studio.

$$\vec{M}_{avg}(t_i) = \sum_{k=i-r+1}^{i} w(t_k).\overrightarrow{XY}(t_k) \div \sum_{k=i-r+1}^{i} w(t_k)$$

During the moving filtering, the missing data were approximated using the nearest neighbour interpolation method in order to preserve saccades steepness (which would not have been possible with a linear interpolation). The distance between two successive data samples was computed as follows:

$$D(t_i) = \left\| \vec{M}_{avg}(t_{i+r}) - \vec{M}_{avg}(t_{i-1}) \right\|$$

During the saccades (ballistic eye movement), several data samples could have a distance above the threshold. Similar to what is performed in Tobii studio, if the data

samples belonged to a temporal window of less than 6 samples, only the highest peak was conserved.

$$saccades = \left\{ t_i, \quad \begin{cases} & D(t_i) > h \\ \forall k \neq i, & |i-k| < 6 \Rightarrow \ D(t_i) > D(t_k) \end{cases} \right\}$$

Segments of data samples between saccades or missing data were analyzed as fixations if they contained a series of more than 6 consecutive temporal samples of data once the physiological blinks were removed. The physiological blinks occurring during this temporal window are included in the fixation duration except if they were at the beginning or the end of the fixation period.
