# Supplementary material for: A Novel Analog Reasoning Paradigm: New Insights in Intellectually Disabled Patients
Source: PLoS One. 2016 Feb 26;11(2):e0149717. doi: 10.1371/journal.pone.0149717 (PMC4771701; doi:10.1371/journal.pone.0149717)
Supplement: S1 Table — (DOC) [file pone.0149717.s016.doc]

S1 Table: Normative data of the RT in healthy controls (both children and adults)
