# Supplementary material for: A Novel Analog Reasoning Paradigm: New Insights in Intellectually Disabled Patients
Source: PLoS One. 2016 Feb 26;11(2):e0149717. doi: 10.1371/journal.pone.0149717 (PMC4771701; doi:10.1371/journal.pone.0149717)
Supplement: S3 Table — (DOC) [file pone.0149717.s018.doc]

S3 Table: Pearson's product-moment correlation between the 4 different runs for each group

| **Adult Healthy controls** | RUN1 | RUN2 | RUN3 | RUN4 |
| --- | --- | --- | --- | --- |
| RUN1 |  | 0.88 | 0.86 | 0.81 |
| RUN2 |  |  | 0.84 | 0.79 |
| RUN3 |  |  |  | 0.90 |
| **DS patients** | RUN1 | RUN2 | RUN3 | RUN4 |
| RUN1 |  | 0.91 | 0.90 | 0.55 |
| RUN2 |  |  | 0.92 | 0.70 |
| RUN3 |  |  |  | 0.54 |
| **FraX patients** | RUN1 | RUN2 | RUN3 | RUN4 |
| RUN1 |  | 0.87 | 0.64 | 0.86 |
| RUN2 |  |  | 0.86 | 0.88 |
| RUN3 |  |  |  | 0.83 |
